# Exploring the potential mechanism of atrazine-induced dopaminergic neurotoxicity based on integration strategy

**DOI:** 10.1265/ehpm.24-00079

**Published:** 2024-09-04

**Authors:** Ling Qi, Jingran Yang, Jianan Li

**Affiliations:** Department of Occupational and Environmental Health, College of Public Health, Xuzhou Medical University, 209 Tongshan Road, Yun Long District, Xuzhou 221000, China

**Keywords:** Atrazine, Dopaminergic neurotoxicity, PC12 cells, Environmental exposures, Network toxicology

## Abstract

**Background:**

Atrazine (ATR), a commonly used herbicide, is linked to dopaminergic neurotoxicity, which may cause symptoms resembling Parkinson’s disease (PD). This study aims to reveal the molecular regulatory networks responsible for ATR exposure and its effects on dopaminergic neurotoxicity based on an integration strategy.

**Methods:**

Our approach involved network toxicology, construction of protein-protein interaction (PPI) networks, gene ontology (GO), and Kyoto Encyclopedia of Genes and Genomes (KEGG) pathway analysis, as well as molecular docking techniques. Subsequently, we validated the predicted results in PC12 cells *in vitro*.

**Results:**

An integrated analysis strategy indicating that 5 hub targets, including mitogen-activated protein kinase 3 (Mapk3), catalase (Cat), heme oxygenase 1 (Hmox1), tumor protein p53 (Tp53), and prostaglandin-endoperoxide synthase 2 (Ptgs2), may play a crucial role in ATR-induced dopaminergic injury. Molecular docking indicated that the 5 hub targets exhibited certain binding activity with ATR. Cell counting kit-8 (CCK8) results illustrated a dose-response relationship in PC12 cells. Real-time quantitative polymerase chain reaction (RT-qPCR) displayed notable changes in the expression of hub targets mRNA levels, with the exception of Mapk3. Western blotting results suggested that ATR treatment in PC12 cells resulted in an upregulation of the Cat, Hmox1, and p-Mapk3 protein expression levels while causing a downregulation in Tp53, Ptgs2, and Mapk3.

**Conclusion:**

Our findings indicated that 5 hub targets identified could play a vital role in ATR-induced dopaminergic neurotoxicity in PC12 cells. These results provide preliminary support for further investigation into the molecular mechanism of ATR-induced toxicity.

**Supplementary information:**

The online version contains supplementary material available at https://doi.org/10.1265/ehpm.24-00079.

## Graphical Abstract

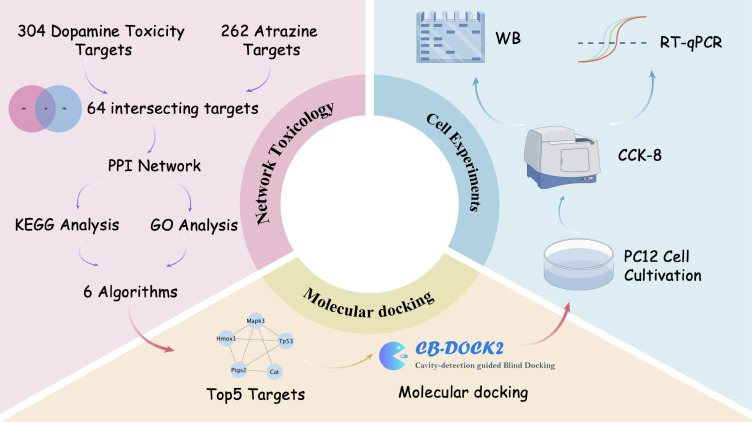

## 1. Introduction

Atrazine (ATR), an s-triazine herbicide, is employed for weed control in crops [1]. ATR continues to persist in the environment due to its long half-life and slow degradation rate from extensive agricultural use, leading to significant residues [2]. It was found that agricultural workers had a peak concentration of 10.4 ng/ml of ATR mercapturate in their urine samples at the end of the workday [3]. Moreover, exposure to ATR has been found to pose risks to various aspects of human health, including the nervous system [4], reproductive development [5], immune system [6], and more. Therefore, it is evident that the substantial harm caused by ATR cannot be underestimated.

Exposure to ATR during neurodevelopment can impact the developing nervous system, particularly harming the dopaminergic system [7]. The relevant study showed that ATR could downregulate tyrosine hydroxylase and dopamine transporter mRNA in the midbrain, potentially leading to decreased dopamine content in the striatum [8]. Decreased dopamine levels and dopaminergic neuron injury are associated with Parkinson’s disease (PD), with investigations indicating a potential connection between ATR exposure and PD [9]. Despite this, the underlying mechanism of ATR-induced dopaminergic injury remains unclear, necessitating further investigation.

In the context of rapid advances in systems biology, integrating network toxicology with molecular docking is a promising strategy. The fundamental principle of network toxicology involves the identification of biological networks that disease features and chemical targets [10], elucidating complex biological mechanisms through extensive datasets [11]. Meanwhile, molecular docking, a recognized tool for investigating dynamic molecular information at the molecular level, probes chemical bonds, specific binding sites, spatial structural changes, and binding energy of toxicants and proteins [12]. Combined with network toxicology and molecular docking has become a potentially effective strategy for unveiling potential molecular mechanisms in toxicity prediction.

This study aims to preliminarily clarify the mechanism of ATR-induced dopaminergic injury by investigating mechanisms using network toxicology and molecular docking, coupled with *in vitro* cell experiments. This study can provide additional evidence for relevant studies on ATR exposure and provide a reference for further toxicological studies.

## 2. Materials and methods

### 2.1 Predicting ATR-associated targets

We acquired the 3D structure of ATR from the PubChem database (https://pubchem.ncbi.nlm.nih.gov/) and uploaded to the SwissTarget-Prediction (http://www.swisstargetprediction.ch), Comparative Toxicogenomics Database (CTD, http://ctdbase.org/), and STITCH database (http://stitch.embl.de/) for the prediction of potential targets associate with ATR. For all 3 databases, the species selection was set to *Rattus norvegicus*, while other remaining parameters were maintained at default values.

### 2.2 Screening targets for dopaminergic neurotoxicity

The CTD database (http://ctdbase.org/) was employed to identify potential targets associated with dopaminergic neurotoxicity in rats. Keywords including “Dopamine injurys”, “Dopamine neurons injurys”, “Dopamine neurons toxicity”, “Dopamine toxicity”, “Dopaminergic injurys”, “Dopaminergic neurons injurys”, “Dopaminergic neurons toxicity”, and “Dopaminergic toxicity” were used for search. *Rattus norvegicus* was specifically chosen as the species.

### 2.3 Venn analysis for intersection targets

Venny 2.1.0 (http://bioinfo.cnb.csic.es/tools/venny/index.html) was used to obtain the intersection targets between the dopaminergic injury-related targets and predicted ATR targets. Subsequently, Venn diagrams were established for visualization, and the intersections were considered as potential targets of dopaminergic neurotoxicity triggered by ATR.

### 2.4 Constructing protein-protein interaction (PPI) network

The intersecting targets were imported into STRING database (http://string-db.org), specifying restricted species as *Rattus norvegicus*. We set a minimum interaction score threshold of 0.4 and visualized the interaction network of potential hub targets using Cytoscape 3.10.0 following previous study [13]. Subsequently, we conducted a systematic analysis of network parameters.

### 2.5 Gene ontology (GO) and Kyoto encyclopedia of genes and genomes (KEGG) pathways analysis

We used Metascape database (https://metascape.org/) to conduct an enrichment analysis of Gene Ontology (GO) and Kyoto Encyclopedia of Genes and Genomes (KEGG) pathways for the obtained intersecting targets. For analysis, *Rattus norvegicus* was selected as the species and a significance cut-off of *p* < 0.05 was applied. The top 10 GO terms in biological process (BP), cellular component (CC), and molecular functions (MF) as well as a total of 17 KEGG pathways were finally presented in a bar and bubble graph.

### 2.6 Calculation of hub targets

The top 10 hub targets were calculated using Edge Percolated Component (EPC), Density of Maximum Neighborhood Component (MNC), Maximal Clique Centrality (MCC), as well as Closeness, Radiality, and Betweenness methods of cytoHubba. Intersection targets obtained from the 6 algorithms function as further experimental validation objects.

### 2.7 Molecular docking

For molecular docking, 3D chemical structure of ATR was obtained from PubChem database in SDF file format. 3D crystal structures of hub targets proteins were obtained in PDB format from RCSB Protein Data Bank (https://www.rcsb.org/). Molecular docking analysis was conducted using CB-Dock2 server (https://cadd.labshare.cn/cb-dock2/php/index.php) to determine the binding affinity between ATR and hub proteins. CB-Dock2 server generated multiple models of complex structures, and the initial complex structure was chosen for further investigation based on both the number of binding pockets and the docking score [14]. Binding energy provides a quantitative measure of potential binding between hub proteins and chemicals. According to consensus, binding energies lower than −4.25 kcal/mol imply certain binding activity, while energies below −5.0 kcal/mol indicate good binding, and energies under −7.0 kcal/mol suggest strong binding between proteins and chemicals [15].

### 2.8 Cell culture and treatment

PC12 cells were obtained from Shanghai Guandao Biological Engineering Co., Ltd (Shanghai, China), cultured in high-glucose Dulbecco’s modified Eagle’s medium (DMEM) (Hyclone, Logan, UT, USA) supplemented with 10% fetal bovine serum (FBS) (ScienCell, Carlsbad, CA, USA) and 1% double antibodies (HyClone). Cells were cultured at 37 °C in a humidified atmosphere containing 5% CO2 and 95% air. They were sub-cultured after 72 hours. ATR (97%) was procured from Beijing Solarbio Technology Co., Ltd (Beijing, China). ATR solutions for cell treatment were prepared at various concentrations (200, 400, 600, 800, and 1000 µM) by dissolving in DMSO (MP Biomedicals, OH, USA), ensuring a final DMSO concentration below 0.1%. A control medium containing 0.1% DMSO served as the vehicle control (0 mM).

### 2.9 Cell viability assay

Cell Counting Kit-8 (CCK8) (Biosharp, Hefei, China) was used to evaluate cell viability. PC12 cells seeded in 96-well plates were treated with gradient ATR (0, 200, 400, 600, 800, and 1000 µM) for 72 hours. Following the manufacturer’s instructions, we added 10 µL of CCK8 reagent to each well. We measured the absorbance at 450 nm after an incubation of 2 hours at 37 °C.

### 2.10 Real-time quantitative polymerase chain reaction (RT-qPCR)

We exposed PC12 cells to 0, 100, 200, and 400 µM ATR for 72 hours. Then we extracted the total RNA using the RNA Isolation Kit (Vazyme, Nanjing, China). We reverse transcribed the total RNA into cDNA according to the manufacturer’s instructions (Vazyme). The primers, designed and synthesized by Wuhan Servicebio Technology Co., Ltd. (Wuhan, China), are detailed in Table S1. The procedure followed normal reaction conditions described in our previous study [16]. The 2^−ΔΔCt^ method was used to calculate relative mRNA expression levels.

### 2.11 Western blotting

PC12 cells treated with a gradient were harvested and lysed in a lysis buffer containing RIPA, PMSF, with a phosphatase inhibitor. Protein concentrations were measured using the BCA kit (Beyotime, Shanghai, China). Primary antibody was selected as Gapdh (Abways technology, Shanghai, China), catalase (Cat) (Wanlei bio, Shenyang, China), tumor protein p53 (Tp53) (Proteintech, Rosemont, IL, USA), Mitogen-Activated Protein Kinase 3 (Mapk3) (Wanlei bio), p-Mapk3 (Proteintech), Heme Oxygenase 1 (Hmox1) (Wanlei bio), prostaglandin-endoperoxide synthase 2 (Ptgs2). The primary antibodies were diluted 1:1000 in blocking buffer and membranes were incubated overnight at 4 °C in the primary antibodies. The following day, the membranes were incubated for 1 hour at 24–26 °C with the chosen secondary antibody, which was HRP-conjugated goat anti-rabbit IgG (Wanlei Bio), diluted at 1:5000 in blocking buffer. Finally, the membrane with super sensitive ECL chemiluminescence (Beyotime) method was detected. The detailed procedure followed the descriptions in the previous study [17].

### 2.12 Statistical analysis

All the results in this investigation were analyzed by using SPSS 28.0 (SPSS, Chicago, IL, USA) statistical software. Data are presented as the mean ± standard error of the mean (SEM). We used one-way analysis of variance (ANOVA) for all datasets and conducted multiple group comparisons using Dunnett’s multiple tests, with significance indicated for *p*-values less than 0.05 (*p* < 0.05).

## 3. Results

### 3.1 Candidate targets screen and construction of PPI network

**Fig. 1:**
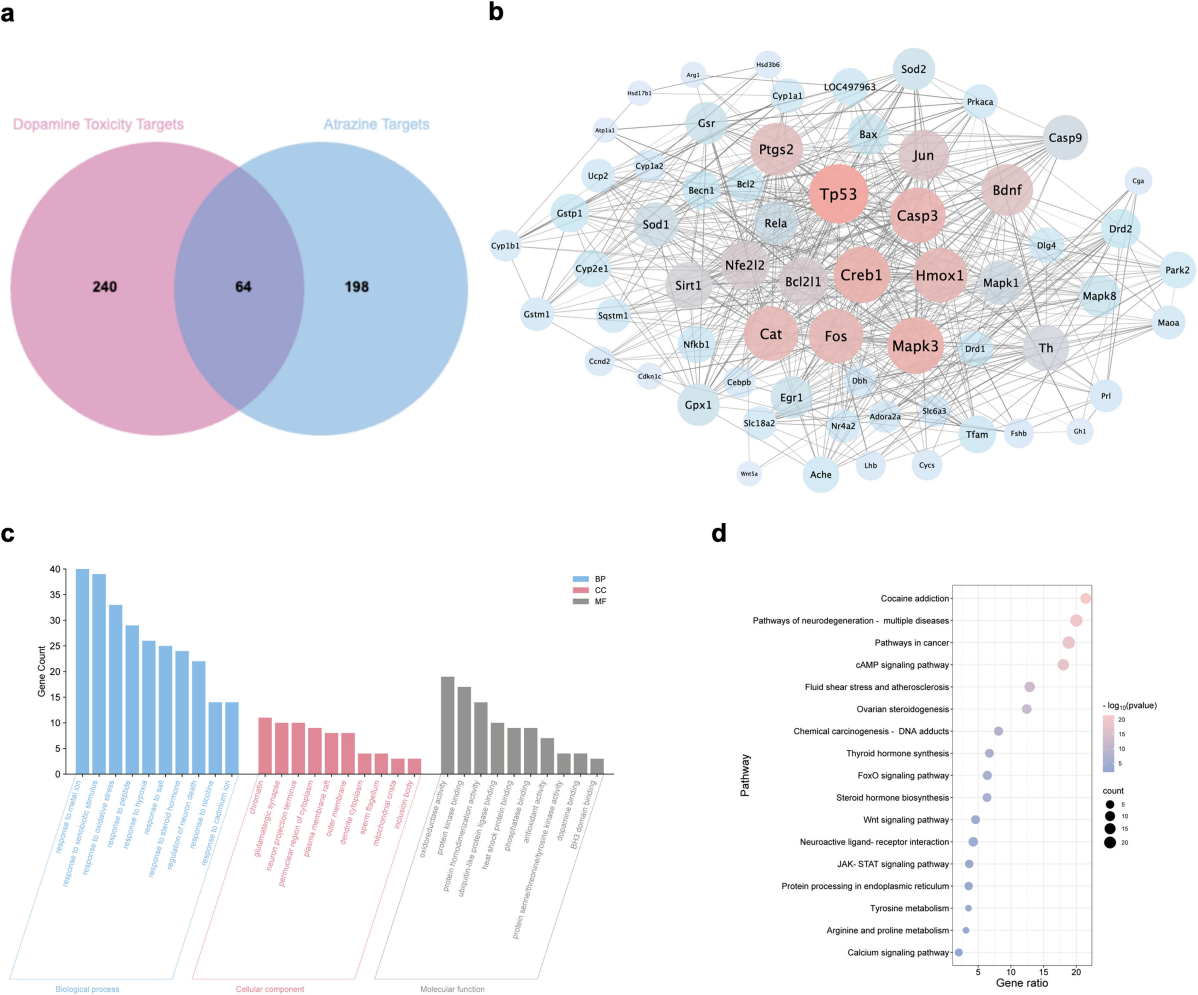
Venn diagram, PPI network, and enrichment analysis. (a) Venn diagram showing the overlap between ATR-related targets and dopamine neurotoxicity-related targets. (b) PPI network construction. The thickness of edges represents the combined score, while node color and size indicate the degree of interaction. (c) GO enrichment analysis. (d) KEGG pathway enrichment analysis.

After eliminating duplicates, a total of 262 ATR targets acting on rats were identified. Simultaneously, 304 targets associated with dopaminergic neurotoxicity in rats were obtained from the CTD database. Among all the captured data, there were 64 common targets at the intersection of dopaminergic injury and ATR targets (Fig. 1a). These intersecting targets were then utilized to construct a PPI network in the STRING database (Fig. 1b, Table S2). The PPI network comprised 64 nodes and 662 edges, exhibiting an average node degree of 20.7.

### 3.2 GO and KEGG enrichment analyses

GO enrichment analysis was conducted as shown in Fig. 1c and Table S3. The BP enrichment analysis primarily included “response to metal ion”, “response to xenobiotic stimulus”, and “response to oxidative stress”. The CC enrichment analysis primarily comprised “chromatin”, “glutamatergic synapse”, and “neuron projection terminus”. The MF enrichment analysis primarily comprised “oxidoreductase activity”, “protein kinase binding”, and “protein homodimerization activity”.

KEGG pathway analysis was carried out as depicted in Fig. 1d and Table S4. The intersecting targets were predominantly enriched in pathways related to “cocaine addiction”, “neurodegeneration in multiple diseases”, and “cancer pathways”.

### 3.3 Screening hub targets and performing molecular docking

**Fig. 2:**
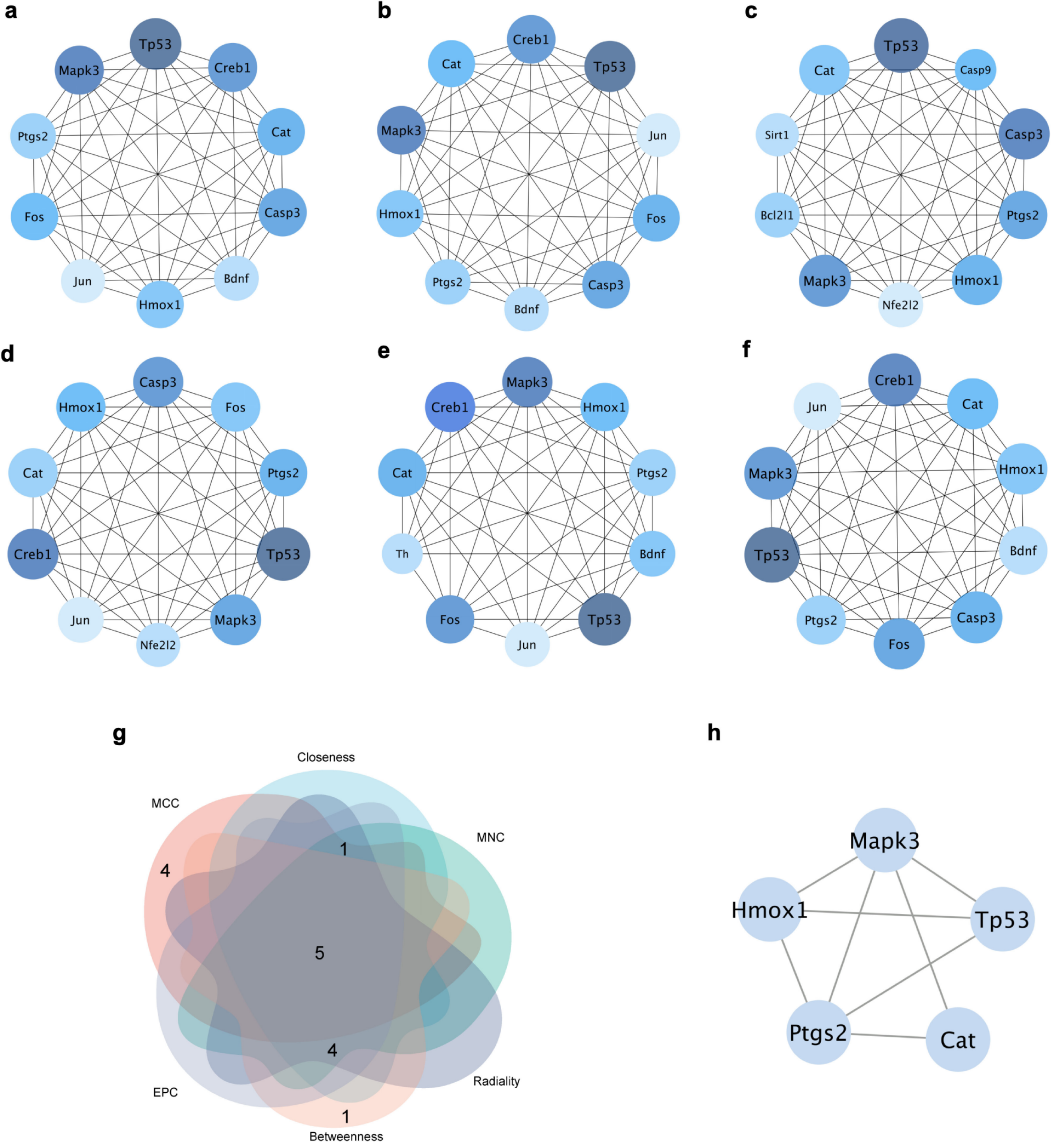
Screening of hub targets. The top 10 hub targets calculated by (a) MNC, (b) closeness, (c) MCC, (d) EPC, (e) betweenness, and (f) radiality. Node color indicates the combined score. (g) Venn diagram of the top 5 hub targets. (h) Construction of the PPI network for the 5 intersecting targets.

**Fig. 3:**
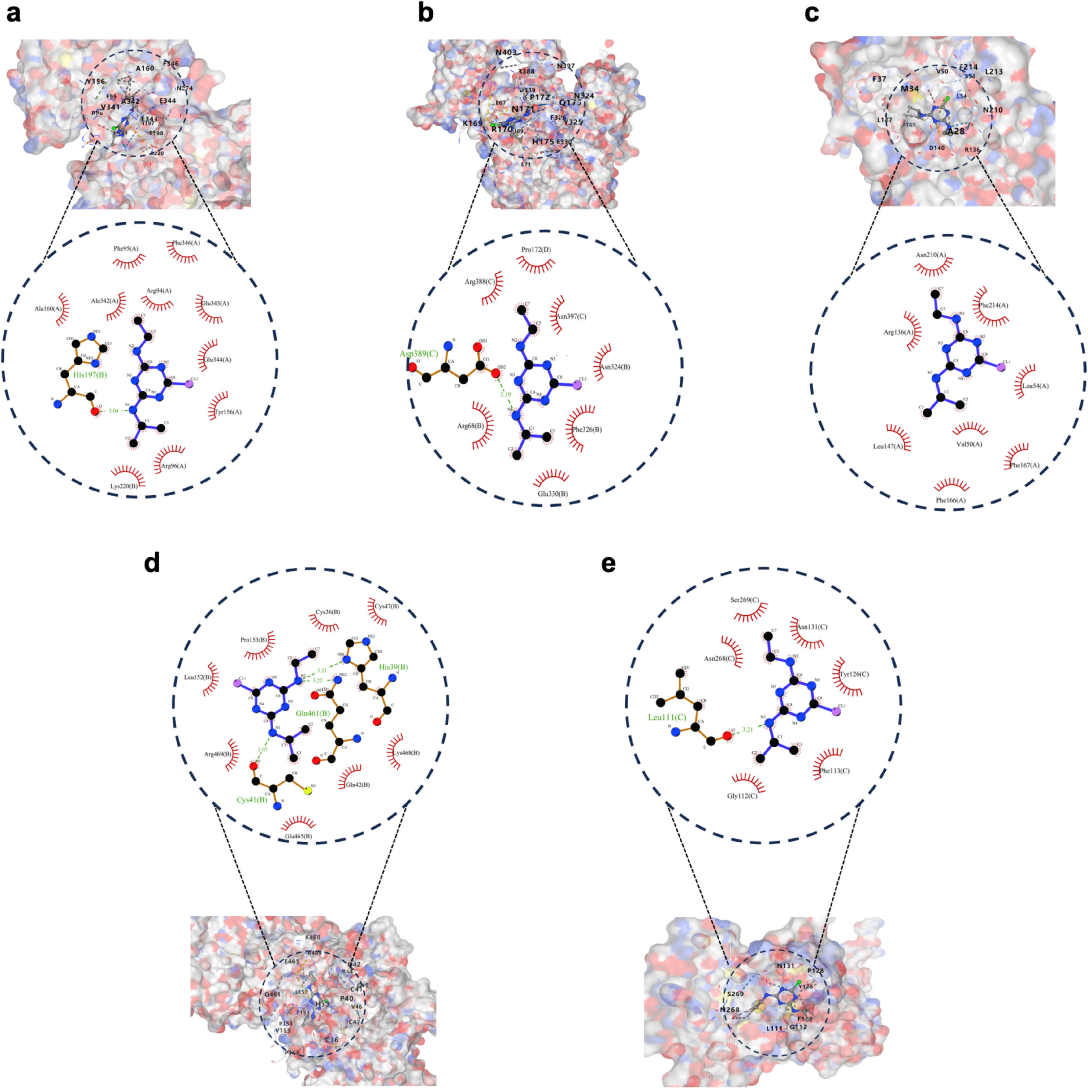
Molecular docking analysis of ATR to 5 hub targets. (a) ATR-Mapk3. (b) ATR-Cat. (c) ATR-Hmox1. (d) ATR-Ptgs2. (e) ATR-Tp53.

The hub targets were selected by calculating the PPI network using 6 algorithms in the cytoHubba plugin of Cytoscape 3.10.0 software (Fig. 2a–f, Tables S5–S10). Finally, the 5 common hub targets were screened, including Tp53, Cat, Ptgs2, Mapk3, and Hmox1. Molecular docking analysis was conducted between ATR and hub targets (Fig. 3, Table S11). Binding energies between ATR and hub targets, except ATR-Tp53, were lower than −5.00 kcal/mol, indicating good binding activity. ATR-Tp53 exhibited a binding energy of −4.60 kcal/mol, slightly below −4.25 kcal/mol, suggesting certain binding activity.

### 3.4 Cytotoxic effects of ATR on PC12 cells

**Fig. 4:**
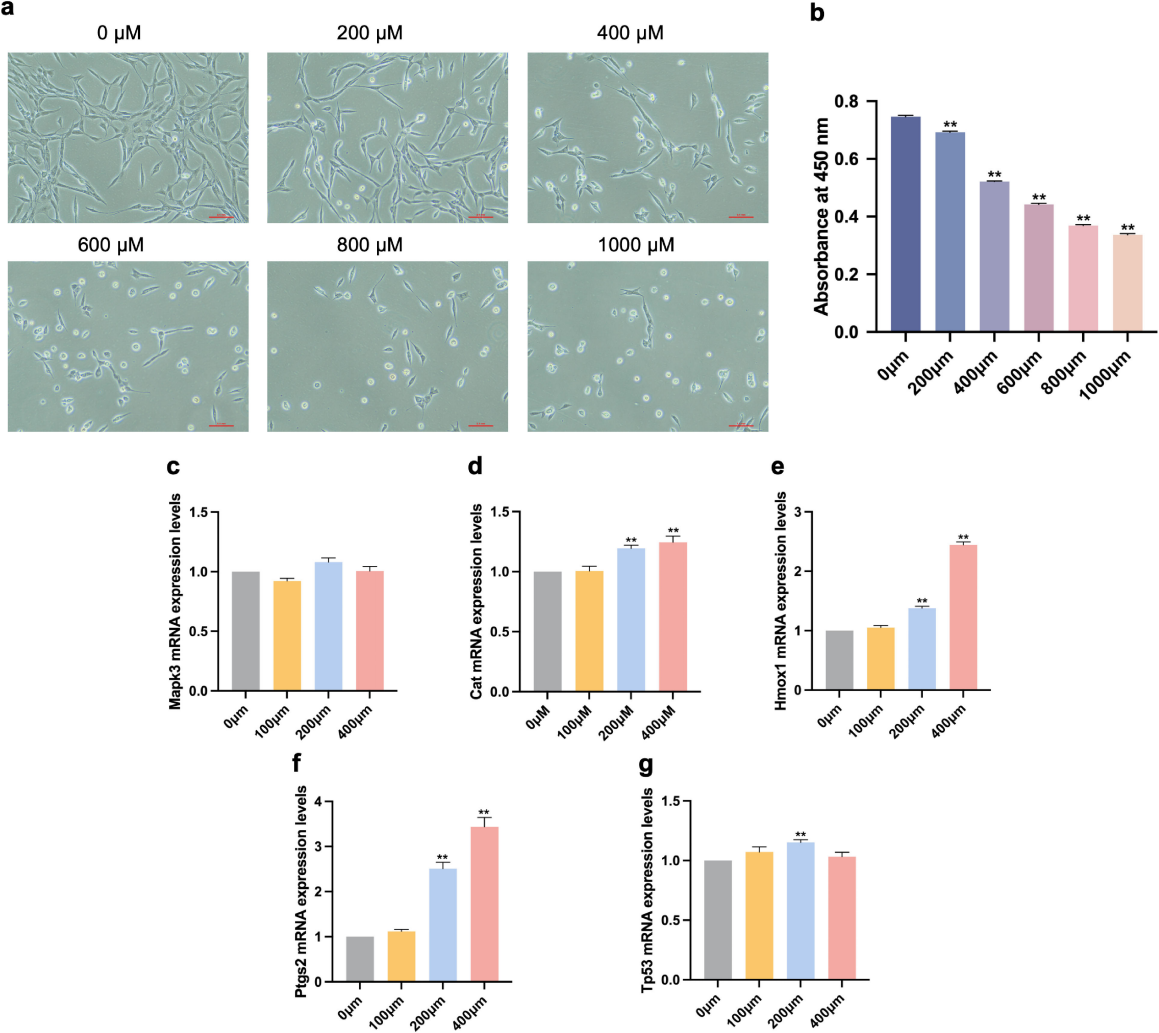
Effects of ATR exposure on cytotoxicity and mRNA expression levels. (a) microscopic images depicting morphological changes under bright-field illumination (b) cell viability was detected using the CCK-8 assay. Each column represents the mean ± S.E.M, n = 6. Effects of ATR on (c) Mapk3, (d) Cat, (e) Hmox1, (f) Ptgs2, (g) Tp53 mRNA expression levels of hub targets in PC12 cells. Expression levels were normalized to Gapdh. Each column represents the mean ± SEM, with n = 9 samples. **p < 0.01 *vs.* 0 µM.

Following treatment with various ATR concentrations for 72 hours, PC12 cells viability was assessed using CCK8. We observed an increased reduction in cell numbers and cell shrinkage corresponding to elevated ATR concentration (Fig. 4a, b). A dose-dependent decline in PC12 cell viability was exhibited by ATR, with a calculated IC50 of 423.88 µM after the 72 hours exposure period (*p* < 0.01).

### 3.5 Effects of ATR on mRNA expression levels of hub targets

After ATR treatment, the relative mRNA expression levels of Mapk3, Cat, Hmox1, Tp53, and Ptgs2 on PC12 cells were examined, as shown in Fig. 4. ATR treatment increased the level of mRNA expression of Cat (0 µM *vs.* 200 µM: *p* < 0.01, 0 µM *vs.* 400 µM: p < 0.01), Hmox1 (0 µM *vs.* 200 µM: *p* < 0.01, 0 µM *vs.* 400-µM: *p* < 0.01) and Ptgs2 (0 µM *vs.* 200 µM: *p* < 0.01, 0 µM *vs.* 400 µM: *p* < 0.01) in the 200 and 400 µM group (Fig. 4d–f). The mRNA expression level of Tp53 only increased in the 200 µM group (0 µM *vs.* 200 µM: *p* < 0.01) (Fig. 4g). Notably, no significant differences were observed in the mRNA expression levels of Mapk3 (*p* = 0.0039) across the treatment groups (Fig. 4c).

### 3.6 Effects of ATR on protein expression levels of hub targets

**Fig. 5:**
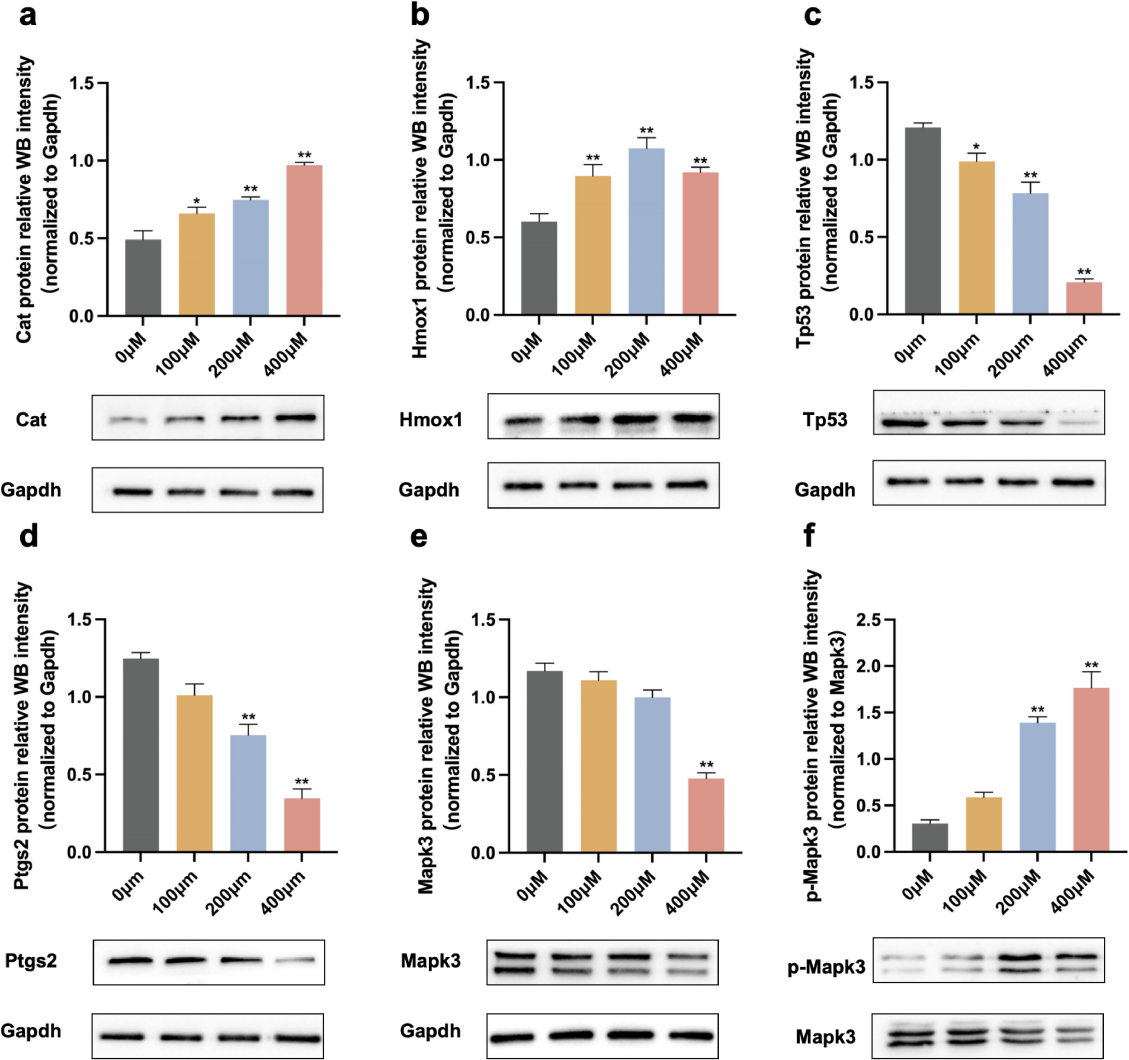
Effects of ATR exposure on protein expression levels. (a) Cat. (b) Hmox1. (c) Tp53. (d) Ptgs2. (e) Mapk3. (f) p-Mapk3. All the expression levels except p-Mapk3 were normalized to Gapdh, p-Mapk3 were normalized Mapk3. Each column represents the mean ± S.E.M, n = 6, *p < 0.05 *vs.* 0 µM, **p < 0.01 *vs.* 0 µM.

The expression of Mapk3, Cat, Hmox1, Tp53, and Ptgs2 protein levels in PC12 cells after ATR exposure are shown in Fig. 5. ATR treatment significantly increased the levels of Cat (*p* < 0.01) and Hmox1 (*p* < 0.01) protein expression in ATR-treated groups (Fig. 5a, b). Tp53 protein expression levels were significantly reduced in the ATR-treated group (*p* < 0.01) (Fig. 5c). Ptgs2 (0 µM *vs.* 200 µM: *p* < 0.01, 0 µM *vs.* 400 µM: *p* < 0.01) protein expression levels decreased in both the 200 µM and 400 µM groups following ATR treatment (Fig. 5d). Mapk3 protein expression levels only decreased in the 400 µM group (0 µM *vs.* 400 µM: *p* < 0.01) (Fig. 5e), while ATR treatment increased the protein expression levels of p-Mapk3 (0 µM *vs.* 200 µM: *p* < 0.01, 0 µM *vs.* 400 µM: *p* < 0.01) in the 200 and 400 µM group (Fig. 5f).

## 4. Discussion

Currently, it is revealed that ATR functions as a toxicant to the nervous system, specifically impacting the dopamine system [18]. However, the underlying mechanism of the neurotoxic effects is still largely undetermined. In this study, network toxicology was employed to predict the hub targets involved in ATR-induced dopaminergic toxicity. Molecular docking was then utilized to model the binding ability between ATR and identified hub targets. Finally, the PC12 cell model was conducted to validate the neurotoxic effects of ATR and the alterations in hub targets. Simultaneously, employing a molecular biology assay, our investigation found that the changes in the mRNA levels of Cat, Hmox1, Ptgs2, and Tp53, along with alterations in the protein levels of 5 hub targets (Mapk3, Cat, Hmox1, Ptgs2, Tp53).

KEGG analysis revealed significant enrichment in neurodegeneration-related pathways. Evidence indicates that ATR exposure can cause injury to multiple brain regions, including dopaminergic and hippocampal damage. Specifically, SD rats exposed to ATR at 10 or 100 mg/kg for 30 days led to hippocampal injury, affecting spatial memory and suggesting a link to neurodegenerative diseases [19]. Our research focuses on dopaminergic injury, a common neurodegenerative condition, suggesting ATR exposure as a significant risk factor for neurodegeneration diseases. Furthermore, GO analysis showed that BP enrichment primarily involved the “response to oxidative stress,” a critical process in cellular damage that could lead to dopaminergic injury. Previous research has demonstrated that ATR exposure induces oxidative stress in the substantia nigra of SD rats treated with 50 mg/kg ATR for 45 days [20], indicating oxidative stress as a crucial factor in ATR-induced dopaminergic injury. Our study also examines changes in Cat expression associated with oxidative stress in this process. CC enrichment analysis focused on “mitochondrial crista,” which enhances cellular respiration and energy production. Electron microscopy revealed degenerative micromorphology in the rat striatum exposed to gradient ATR concentrations for 90 days, revealing decreased mitochondrial cristae and mitochondrial autophagy [21]. This suggests an important alteration in ATR-induced neurological injury. In addition, MF enrichment analysis identified “phosphatase binding” as a key process, essential for regulating various cellular activities. Evidence suggests altered phosphorylation expression in ATR-induced dopaminergic injury, including p-Fos and p-Creb [19]. Our research also investigates changes in p-Mapk3 expression during this process. These KEGG and GO enrichment results offer potential research directions for further exploring ATR-induced dopaminergic neurotoxicity.

The MAPK signaling pathway is crucial for cell proliferation and survival. Mapk3, a key member of the downstream MAPK signaling pathway, regulates various biological processes, by integrating extracellular signals to promote cell proliferation and growth [22]. Additionally, previous research has highlighted the crucial role of the MAPK signaling pathway in PD, in which dysregulation results in harmful effects such as microglia activation, neuroinflammation, oxidative stress, and apoptosis [23]. The MAPK signaling pathway undergoes a cascade phosphorylation process [24], and phosphorylation of Mapk3 is a crucial step in its activation. PD is primarily caused by necrosis of dopaminergic neurons in the substantia nigra, leading to decreased dopamine in the synaptic gap [25]. Moreover, the over-expression of p-Mapk3 has been demonstrated to inhibit dopamine release in PC12 cells [26]. Our study observed an increase in p-Mapk3 protein expression levels due to ATR exposure. Another study demonstrated a time-dependent elevation of p-Mapk3 protein levels in human chorionic carcinoma cell line JEG-3 treated with 10 µM ATR, resulting in disrupted endocrine development [27]. Through the integration of molecular assays and molecular docking, our results showed that Mapk3 is essential in ATR-induced dopaminergic neuro damage.

Cat, an enzyme implicated in mutagenesis and inflammation, is involved in suppressing apoptosis, associated with oxidative stress conditions [28]. A previous study demonstrated that female zebrafish had increased Cat mRNA expression levels in the liver after 14 days of different concentrations of ATR treatment, indicating an enhanced oxidative stress response in the liver [29]. Furthermore, it is shown that administering rats with ATR at a dosage of 400 mg/kg for 3 weeks led to an increase in Cat activity in serum/cardiac tissue, suggesting ATR-induced cardiotoxicity [30]. This suggests that Cat may be a specific target in ATR-induced toxicity. Exposure to 0.3 mM ATR for 24 hours markedly enhances reactive oxygen species (ROS) production and causes oxidative damage in human neuroblastoma SH-SY5Y (SH-SY5Y) cells, leading to increased Cat levels and activity [31]. This indicates a potential connection between dopaminergic damage and ATR exposure. In our research, we observed upregulation of Cat at both the mRNA and protein expression levels following ATR exposure. Cat can be an antioxidant to play a protective role [28], but it is unclear if the increased expression level following ATR treatment serves as a protective mechanism or triggers other cascading damages.

Hmox1, an enzyme engaged in heme breakdown, is linked to cellular processes, including exerting anti-inflammatory and antioxidant effects [32]. It is believed that oxidative stress and inflammation contribute to the neurodegenerative process in PD [33]. Evidence indicated an increase in Hmox1 in dopaminergic neurons and the peripheries of Lewy bodies in the substantia nigra of individuals with PD [34]. Research suggested that Hmox1 may act as a protective factor by inducing a protective response in neurons and glial cells, thus preventing dopaminergic neuron death due to oxidative [35]. Additionally, accumulation of α-synuclein is a common pathological feature in PD [36], and Hmox1 may aid in the proteasomal breakdown of α-synaptic nuclear proteins, preventing harmful aggregation and safeguarding dopaminergic neurons [32, 37]. In this study, an upregulation in Hmox1 protein and mRNA expression levels was noted after ATR exposure. We deduce that Hmox1 concurrently serves as a protective factor against ATR-induced effects in PC12 cells to some extent. However, we still need to further explore because the mechanisms involved are still unclear.

Ptgs2, known as Cox-2, plays a vital role in synthesizing prostaglandins [38], and is essential in neuroinflammation and neuronal degeneration [39]. Our study revealed that exposure to ATR for 72 hours induced an upregulation in Ptgs2 mRNA expression levels, aligning with a previous study showing ATR exposure for 24 hours upregulated Ptgs2 mRNA expression levels in PC12 cells [40]. This effect likely resulted from the inflammatory response triggered by oxidative stress, impacting the gene transcription of multiple oxidative stress-sensitive transcription factors [41]. In addition, we observed a decline in Ptgs2 protein expression levels, but the mechanism for this inconsistency in mRNA and protein expression levels is unclear. We infer that this decrease may be due to the inhibition of the translation process. A similar study demonstrated that curcumin induces the expression level of RNA binding protein CUG triplet repeat RNA-binding protein 2, leading to the inhibition of Ptgs2 mRNA translation [42]. The elucidation of the mechanism responsible for the disparate expression patterns observed in Ptgs2 mRNA and protein levels, attributed to ATR, requires additional investigation. Combining the results of molecular docking and molecular assays, we presume that Ptgs2 is pivotal in the response to ATR-induced dopaminergic injury in PC12 cells.

TP53, beyond its established role as a tumor suppressor gene, is also acknowledged as a significant neuronal pro-apoptotic factor, linking to related proteins associated with neurodegenerative disorders [43]. In our study, dose-dependent elevated levels of Tp53 mRNA expression in ATR-treated PC12 cells were demonstrated. A prior study demonstrated elevated expression levels of Tp53 mRNA were observed in the ventral midbrains of rats following treatment with various concentrations of ATR for 45 days, leading to neurodegenerative damage [44]. In the relative quantification of protein, we found that the expression levels of Tp53 were reduced. This is consistent with another study that demonstrated a decrease in Tp53 protein expression levels in SH-SY5Y cells exposed to 0.3 mM ATR for 6 hours, which subsequently contributed to cytotoxicity [31]. These findings collectively suggest that Tp53 could be a specific target of ATR-induced effects. The inconsistent mechanism underlying the expression of Tp53 mRNA and protein remains unclear, for the reason that alterations in protein concentrations cannot be solely attributed to RNA regulation. Significant adjustments in translation rates and protein degradation were also identified, playing a pivotal role in finely tuning eventual protein concentrations [45]. We infer that the treatment of ATR can affect the processes, ultimately resulting in dopaminergic neurotoxicity.

It is imperative to value the crucial roles of 5 hub targets in ATR-induced dopaminergic injury in PC12 cells. However, certain limitations should be acknowledged within this study. Firstly, our results linked to the identified targets were obtained from databases, indicating that the reliability and precision of predictions hinge on the quality of the data. Furthermore, *in vivo* validations of the underlying mechanisms are imperative to substantiate the reported findings.

## 5. Conclusion

In this study, a comprehensive analysis combining network toxicology and molecular docking was performed to explore the mechanism of ATR-induced dopaminergic injury, and preliminary verifying the possible mechanism using PC12 cells. The results of our investigation offer scientific support for the neurotoxic effects of ATR on the dopaminergic system, establishing a basis for further research.

## Abbreviations

ATR: Atrazine
PD: Parkinson’s disease
PC12 cell: Pheochromocytoma 12 cell
GO: Gene Ontology
KEGG: Kyoto Encyclopedia of Genes and Genomes enrichment
RT-qPCR: Real-time quantitative polymerase chain reaction
CCK8: Cell counting kit-8
Mapk3: Mitogen-activated protein kinase 3
Cat: Catalase
Hmox1: Heme oxygenase 1
Tp53: Tumor protein p53
Ptgs2: Prostaglandin-endoperoxide synthase 2
CTD: Comparative Toxicogenomics Database
BP: Biological process
CC: Cellular component
MF: Molecular functions
EPC: Edge Percolated component
MNC: Density of Maximum Neighborhood Component
MCC: maximal clique centrality
DMEM: High-glucose Dulbecco’s modified Eagle’s medium
SEM: Standard error of the mean
ANOVA: Analysis of Variance
SH-SY5Y cell: Human neuroblastoma SH-SY5Y cells
ROS: Reactive oxygen species

## Supplementary information

**Additional file 1:** **Table S1.** Sequences of primer pairs used in the real-time quantitative PCR reactions. **Table S2.** Information of PPI network. **Table S3.** GO enrichment analysis. **Table S4.** KEGG enrichment analysis. **Table S5.** Top 10 targets calculated by MNC method. **Table S6.** Top 10 targets calculated by Closeness method. **Table S7.** Top 10 targets calculated by MCC method. **Table S8.** Top 10 targets calculated by EPC method. **Table S9.** Top 10 targets calculated by Betweenness method. **Table S10.** Top 10 targets calculated by Radiality method. **Table S11.** Molecular docking.

## Declarations

### Availability of data and material

All data are incorporated into the article and its online supplementary material.
