# Supplementary material for: Exploring the potential mechanism of atrazine-induced dopaminergic neurotoxicity based on integration strategy
Source: Environ Health Prev Med. 2024 Sep 4;29:46. doi: 10.1265/ehpm.24-00079 (PMC11391274; doi:10.1265/ehpm.24-00079)
Supplement: Supplementary file 1 — Additional file 1: Table S1. Sequences of primer pairs used in the real-time quantitative PCR reactions. Table S2. Information of PPI network. Table S3. GO enrichment analysis. Table S4. KEGG enrichment analysis. Table S5. Top 10 targets calculated by MNC method. Table S6. Top 10 targets calculated by Closeness method. Table S7. Top 10 targets calculated by MCC method. Table S8. Top 10 targets calculated by EPC method. Table S9. Top 10 targets calculated by Betweenness method. Table S10. Top 10 targets calculated by Radiality method. Table S11. Molecular docking. [file ehpm-29-046-s001.docx]

Supplementary Material

Exploring the Potential Mechanism of Atrazine-induced Dopaminergic Neurotoxicity Based on Integration Strategy

Ling Qi^a,1^, Jingran Yang^a,1^, Jianan Li^a^

^a^Department of Occupational and Environmental Health, College of Public Health, Xuzhou Medical University, 209 Tongshan Road, Yun Long District, Xuzhou 221000, China.

^1^These authors contributed equally to the article.

* Correspondence:

Jianan Li

lijianan512133@163.com

# Supplementary Tables

**Table S1.** Sequences of primer pairs used in the real-time quantitative PCR reactions

| **Target gene** | **Primer sequences (5'-3')** | **Product length (bp)** |
| --- | --- | --- |
| Gapdh | GGTGGAAGAATGGGAGTTGCT | 138 |
|  | GGTGGAAGAATGGGAGTTGCT |  |
| Tp53 | GTCATCTTCCGTCCCTTCTCAA | 130 |
|  | CTGGCAGAACAGCTTATTGAGG |  |
| Mapk3 | GAGACATCCTCAGAGCACCCA | 216 |
|  | TGTTGATAAGCAGATTGGAGGG |  |
| Ptgs2 | CTGATGACTGCCCAACTCCC | 143 |
|  | CTGGGCAAAGAATGCGAACA |  |
| Hmox1 | CACAGGGTGACAGAAGAGGCT | 109 |
|  | TCTGTGAGGGACTCTGGTCTTTG |  |
| Cat | TCTCCATCAGGTTACTTTCTTGTTC | 150 |
|  | ATGCCCTGGTCAGTCTTGTAATG |  |

**Table S2.** Information of PPI network

| **#node1** | **node2** | **neighborhood_on_chromosome** | **gene_fusion** | **phylogenetic_cooccurrence** | **homology** | **coexpression** | **experimentally_determined_interaction** | **database_annotated** | **automated_textmining** | **combined_score** |
| --- | --- | --- | --- | --- | --- | --- | --- | --- | --- | --- |
| Ache | Ptgs2 | 0 | 0 | 0 | 0 | 0 | 0.054 | 0 | 0.523 | 0.378 |
| Ache | Fos | 0 | 0 | 0 | 0 | 0 | 0 | 0 | 0.496 | 0.235 |
| Ache | Cat | 0 | 0 | 0 | 0 | 0.049 | 0 | 0 | 0.729 | 0.209 |
| Ache | Slc18a2 | 0 | 0 | 0 | 0 | 0.06 | 0 | 0 | 0.43 | 0.227 |
| Ache | Casp3 | 0 | 0 | 0 | 0 | 0 | 0 | 0 | 0.6 | 0.165 |
| Ache | Creb1 | 0 | 0 | 0 | 0 | 0 | 0 | 0 | 0.57 | 0.165 |
| Ache | Hmox1 | 0 | 0 | 0 | 0 | 0 | 0 | 0 | 0.445 | 0.213 |
| Ache | Gsr | 0 | 0 | 0 | 0 | 0 | 0 | 0 | 0.446 | 0.876 |
| Ache | Th | 0 | 0 | 0 | 0 | 0.057 | 0 | 0 | 0.713 | 0.174 |
| Ache | Drd2 | 0 | 0 | 0 | 0 | 0.093 | 0 | 0 | 0.391 | 0.193 |
| Ache | Dbh | 0 | 0 | 0 | 0 | 0 | 0 | 0 | 0.541 | 0.161 |
| Ache | Dlg4 | 0 | 0 | 0 | 0 | 0.064 | 0.082 | 0 | 0.447 | 0.259 |
| Ache | Maoa | 0.044 | 0 | 0 | 0 | 0.06 | 0 | 0 | 0.585 | 0.328 |
| Ache | Gpx1 | 0 | 0 | 0 | 0 | 0 | 0 | 0 | 0.562 | 0.695 |
| Ache | Bdnf | 0 | 0 | 0 | 0 | 0.05 | 0 | 0 | 0.699 | 0.164 |
| Ptgs2 | Sirt1 | 0 | 0 | 0 | 0 | 0 | 0 | 0 | 0.571 | 0.165 |
| Ptgs2 | Sod1 | 0 | 0 | 0 | 0 | 0.059 | 0 | 0 | 0.44 | 0.165 |
| Ptgs2 | Ucp2 | 0 | 0 | 0 | 0 | 0 | 0 | 0 | 0.416 | 0.18 |
| Ptgs2 | Sod2 | 0 | 0 | 0 | 0 | 0.064 | 0 | 0 | 0.434 | 0.202 |
| Ptgs2 | Th | 0 | 0 | 0 | 0 | 0 | 0 | 0 | 0.49 | 0.21 |
| Ptgs2 | Rela | 0 | 0 | 0 | 0 | 0 | 0 | 0 | 0.583 | 0.218 |
| Ptgs2 | Tp53 | 0 | 0 | 0 | 0 | 0 | 0.666 | 0 | 0.906 | 0.222 |
| Fos | Sirt1 | 0 | 0 | 0 | 0 | 0 | 0.217 | 0 | 0.553 | 0.229 |
| Fos | Nfe2l2 | 0 | 0 | 0 | 0 | 0.057 | 0.181 | 0.297 | 0.344 | 0.313 |
| Fos | Mapk1 | 0 | 0 | 0 | 0 | 0 | 0.321 | 0.962 | 0.728 | 0.379 |
| Fos | Ptgs2 | 0 | 0 | 0 | 0 | 0.064 | 0 | 0 | 0.683 | 0.42 |
| Fos | Fshb | 0 | 0 | 0 | 0 | 0.057 | 0 | 0 | 0.464 | 0.45 |
| Fos | Slc18a2 | 0 | 0 | 0 | 0 | 0 | 0 | 0 | 0.412 | 0.491 |
| Fos | Gstp1 | 0 | 0 | 0 | 0 | 0 | 0 | 0.24 | 0.339 | 0.645 |
| Fos | Nfkb1 | 0 | 0 | 0 | 0 | 0.048 | 0.081 | 0.121 | 0.475 | 0.949 |
| Fos | Hmox1 | 0 | 0 | 0 | 0 | 0 | 0 | 0.316 | 0.532 | 0.972 |
| Fos | Prl | 0 | 0 | 0 | 0 | 0 | 0 | 0 | 0.676 | 0.253 |
| Fos | Nr4a2 | 0 | 0 | 0 | 0 | 0.293 | 0.134 | 0 | 0.528 | 0.193 |
| Fos | Lhb | 0 | 0 | 0 | 0 | 0 | 0 | 0 | 0.738 | 0.183 |
| Fos | Tp53 | 0 | 0 | 0 | 0 | 0 | 0.164 | 0 | 0.763 | 0.169 |
| Fos | Prkaca | 0 | 0 | 0 | 0 | 0 | 0.162 | 0.8 | 0.096 | 0.192 |
| Fos | Th | 0 | 0 | 0 | 0 | 0 | 0 | 0 | 0.848 | 0.169 |
| Fos | Rela | 0 | 0 | 0 | 0 | 0 | 0.461 | 0.637 | 0.535 | 0.189 |
| Fos | Mapk8 | 0 | 0 | 0 | 0 | 0 | 0.385 | 0.905 | 0.553 | 0.234 |
| Fos | Mapk3 | 0 | 0 | 0 | 0 | 0 | 0.321 | 0.959 | 0.745 | 0.395 |
| Fos | Jun | 0 | 0 | 0 | 0 | 0.763 | 0.963 | 0.924 | 0.995 | 0.845 |
| Cat | Sirt1 | 0.043 | 0 | 0 | 0 | 0.061 | 0 | 0 | 0.786 | 0.582 |
| Cat | Tfam | 0 | 0 | 0 | 0 | 0.059 | 0 | 0 | 0.595 | 0.334 |
| Cat | Nfe2l2 | 0 | 0 | 0 | 0 | 0.079 | 0 | 0 | 0.836 | 0.451 |
| Cat | Mapk1 | 0 | 0 | 0 | 0 | 0 | 0.082 | 0 | 0.407 | 0.656 |
| Cat | Sod1 | 0.096 | 0 | 0 | 0 | 0.172 | 0.528 | 0.952 | 0.926 | 0.207 |
| Cat | Ptgs2 | 0 | 0 | 0 | 0 | 0.046 | 0.193 | 0 | 0.719 | 0.171 |
| Cat | Fos | 0 | 0 | 0 | 0 | 0.059 | 0 | 0 | 0.514 | 0.155 |
| Cat | Cyp1a2 | 0.044 | 0 | 0 | 0 | 0.099 | 0 | 0 | 0.376 | 0.177 |
| Cat | Cycs | 0 | 0 | 0 | 0 | 0.137 | 0.082 | 0 | 0.356 | 0.221 |
| Cat | Park2 | 0 | 0 | 0 | 0 | 0 | 0.133 | 0 | 0.39 | 0.869 |
| Cat | Creb1 | 0 | 0 | 0 | 0 | 0 | 0 | 0 | 0.449 | 0.246 |
| Cat | Th | 0 | 0 | 0 | 0 | 0.059 | 0 | 0 | 0.467 | 0.171 |
| Cat | Maoa | 0 | 0 | 0 | 0 | 0.063 | 0 | 0 | 0.479 | 0.162 |
| Cat | Cyp1a1 | 0.044 | 0 | 0 | 0 | 0.079 | 0 | 0 | 0.505 | 0.188 |
| Cat | Jun | 0 | 0 | 0 | 0 | 0 | 0 | 0 | 0.561 | 0.312 |
| Cat | Gstm1 | 0 | 0 | 0 | 0 | 0.059 | 0.161 | 0 | 0.49 | 0.356 |
| Cat | Gstp1 | 0 | 0 | 0 | 0 | 0.049 | 0.161 | 0 | 0.535 | 0.46 |
| Cat | Ucp2 | 0 | 0 | 0 | 0 | 0.057 | 0 | 0 | 0.616 | 0.241 |
| Cat | LOC497963 | 0 | 0 | 0 | 0 | 0 | 0 | 0 | 0.643 | 0.257 |
| Cat | Mapk3 | 0 | 0 | 0 | 0 | 0 | 0.082 | 0 | 0.696 | 0.153 |
| Cat | Tp53 | 0 | 0 | 0 | 0 | 0 | 0 | 0 | 0.739 | 0.151 |
| Cat | Cyp2e1 | 0.044 | 0 | 0 | 0 | 0.173 | 0 | 0 | 0.734 | 0.226 |
| Cat | Hmox1 | 0 | 0 | 0 | 0 | 0.062 | 0.084 | 0 | 0.894 | 0.265 |
| Cat | Gpx1 | 0 | 0 | 0 | 0 | 0.193 | 0.47 | 0 | 0.93 | 0.296 |
| Cat | Gsr | 0 | 0 | 0 | 0 | 0.34 | 0.328 | 0 | 0.958 | 0.154 |
| Cat | Sod2 | 0.084 | 0 | 0 | 0 | 0.081 | 0.329 | 0.952 | 0.904 | 0.234 |
| Slc18a2 | Slc6a3 | 0 | 0 | 0 | 0 | 0.092 | 0.164 | 0 | 0.819 | 0.239 |
| Slc18a2 | Th | 0 | 0 | 0 | 0 | 0.124 | 0.413 | 0 | 0.937 | 0.152 |
| Casp3 | Sirt1 | 0 | 0 | 0 | 0 | 0 | 0 | 0 | 0.715 | 0.156 |
| Casp3 | Tfam | 0 | 0 | 0 | 0 | 0 | 0 | 0 | 0.522 | 0.163 |
| Casp3 | Nfe2l2 | 0 | 0 | 0 | 0 | 0 | 0.065 | 0 | 0.697 | 0.164 |
| Casp3 | Mapk1 | 0 | 0 | 0 | 0 | 0.049 | 0.12 | 0.859 | 0.534 | 0.177 |
| Casp3 | Sod1 | 0 | 0 | 0 | 0 | 0 | 0.092 | 0 | 0.614 | 0.184 |
| Casp3 | Ptgs2 | 0 | 0 | 0 | 0 | 0 | 0 | 0 | 0.754 | 0.624 |
| Casp3 | Fos | 0 | 0 | 0 | 0 | 0 | 0 | 0 | 0.624 | 0.165 |
| Casp3 | Cat | 0 | 0 | 0 | 0 | 0 | 0 | 0 | 0.801 | 0.237 |
| Casp3 | Jun | 0 | 0 | 0 | 0 | 0 | 0.077 | 0 | 0.732 | 0.474 |
| Casp3 | Egr1 | 0 | 0 | 0 | 0 | 0 | 0.046 | 0 | 0.491 | 0.154 |
| Casp3 | Ccnd2 | 0 | 0 | 0 | 0 | 0 | 0.044 | 0 | 0.494 | 0.167 |
| Casp3 | Nfkb1 | 0 | 0 | 0 | 0 | 0 | 0.103 | 0.141 | 0.412 | 0.156 |
| Casp3 | Ucp2 | 0 | 0 | 0 | 0 | 0 | 0 | 0 | 0.522 | 0.16 |
| Casp3 | Mapk8 | 0 | 0 | 0 | 0 | 0 | 0.081 | 0 | 0.537 | 0.159 |
| Casp3 | Dlg4 | 0 | 0 | 0 | 0 | 0 | 0.045 | 0 | 0.571 | 0.155 |
| Casp3 | Cyp2e1 | 0 | 0 | 0 | 0 | 0 | 0 | 0 | 0.587 | 0.275 |
| Casp3 | Sod2 | 0 | 0 | 0 | 0 | 0.051 | 0 | 0 | 0.605 | 0.775 |
| Casp3 | Cycs | 0 | 0 | 0 | 0 | 0.064 | 0.126 | 0.25 | 0.453 | 0.181 |
| Casp3 | LOC497963 | 0 | 0 | 0 | 0 | 0 | 0 | 0 | 0.62 | 0.189 |
| Casp3 | Th | 0 | 0 | 0 | 0 | 0 | 0 | 0 | 0.626 | 0.212 |
| Casp3 | Gsr | 0 | 0 | 0 | 0 | 0 | 0 | 0 | 0.629 | 0.226 |
| Casp3 | Rela | 0 | 0 | 0 | 0 | 0 | 0 | 0.324 | 0.528 | 0.298 |
| Casp3 | Sqstm1 | 0 | 0 | 0 | 0 | 0 | 0.143 | 0 | 0.635 | 0.374 |
| Casp3 | Creb1 | 0 | 0 | 0 | 0 | 0 | 0 | 0 | 0.699 | 0.406 |
| Casp3 | Gpx1 | 0 | 0 | 0 | 0 | 0 | 0 | 0 | 0.719 | 0.673 |
| Casp3 | Hmox1 | 0 | 0 | 0 | 0 | 0 | 0 | 0 | 0.812 | 0.402 |
| Casp3 | Tp53 | 0 | 0 | 0 | 0 | 0.058 | 0.085 | 0 | 0.919 | 0.201 |
| Casp3 | Mapk3 | 0 | 0 | 0 | 0 | 0 | 0.12 | 0.859 | 0.812 | 0.236 |
| Casp3 | Casp9 | 0 | 0 | 0 | 0.83 | 0 | 0.707 | 0.959 | 0.946 | 0.156 |
| Creb1 | Sirt1 | 0 | 0 | 0 | 0 | 0.06 | 0.099 | 0 | 0.736 | 0.526 |
| Creb1 | Tfam | 0 | 0 | 0 | 0 | 0.063 | 0 | 0 | 0.521 | 0.41 |
| Creb1 | Nfe2l2 | 0 | 0 | 0 | 0 | 0 | 0.055 | 0.329 | 0.449 | 0.258 |
| Creb1 | Mapk1 | 0 | 0 | 0 | 0 | 0 | 0.087 | 0.839 | 0.631 | 0.292 |
| Creb1 | Ptgs2 | 0 | 0 | 0 | 0 | 0.049 | 0 | 0 | 0.624 | 0.369 |
| Creb1 | Fshb | 0 | 0 | 0 | 0 | 0 | 0 | 0 | 0.439 | 0.232 |
| Creb1 | Fos | 0 | 0 | 0 | 0 | 0 | 0.159 | 0.584 | 0.893 | 0.506 |
| Creb1 | Jun | 0 | 0 | 0 | 0 | 0 | 0.259 | 0.615 | 0.902 | 0.154 |
| Creb1 | LOC497963 | 0 | 0 | 0 | 0 | 0 | 0 | 0.443 | 0.22 | 0.173 |
| Creb1 | Mapk8 | 0 | 0 | 0 | 0 | 0.076 | 0 | 0 | 0.397 | 0.205 |
| Creb1 | Prl | 0 | 0 | 0 | 0 | 0 | 0 | 0 | 0.421 | 0.206 |
| Creb1 | Maoa | 0 | 0 | 0 | 0 | 0 | 0 | 0 | 0.424 | 0.22 |
| Creb1 | Dbh | 0 | 0 | 0 | 0 | 0 | 0 | 0 | 0.443 | 0.245 |
| Creb1 | Ucp2 | 0 | 0 | 0 | 0 | 0 | 0 | 0 | 0.478 | 0.248 |
| Creb1 | Rela | 0 | 0 | 0 | 0 | 0.062 | 0 | 0 | 0.491 | 0.265 |
| Creb1 | Nfkb1 | 0 | 0 | 0 | 0 | 0.051 | 0.127 | 0.134 | 0.391 | 0.295 |
| Creb1 | Drd1 | 0 | 0 | 0 | 0 | 0 | 0.054 | 0 | 0.507 | 0.299 |
| Creb1 | Drd2 | 0 | 0 | 0 | 0 | 0 | 0.054 | 0 | 0.541 | 0.325 |
| Creb1 | Th | 0 | 0 | 0 | 0 | 0 | 0 | 0 | 0.673 | 0.334 |
| Creb1 | Nr4a2 | 0 | 0 | 0 | 0 | 0.043 | 0 | 0 | 0.693 | 0.394 |
| Creb1 | Hmox1 | 0 | 0 | 0 | 0 | 0 | 0 | 0.373 | 0.57 | 0.404 |
| Creb1 | Dlg4 | 0 | 0 | 0 | 0 | 0 | 0 | 0 | 0.719 | 0.42 |
| Creb1 | Egr1 | 0 | 0 | 0 | 0 | 0.05 | 0.082 | 0 | 0.759 | 0.42 |
| Creb1 | Prkaca | 0 | 0 | 0 | 0 | 0.058 | 0.164 | 0.913 | 0.376 | 0.43 |
| Creb1 | Tp53 | 0 | 0 | 0 | 0 | 0 | 0.478 | 0 | 0.919 | 0.461 |
| Creb1 | Mapk3 | 0 | 0 | 0 | 0 | 0 | 0.087 | 0.82 | 0.801 | 0.472 |
| Hmox1 | Sirt1 | 0 | 0 | 0 | 0 | 0 | 0 | 0 | 0.714 | 0.482 |
| Hmox1 | Tfam | 0 | 0 | 0 | 0 | 0 | 0 | 0 | 0.52 | 0.499 |
| Hmox1 | Nfe2l2 | 0 | 0 | 0 | 0 | 0.083 | 0 | 0.397 | 0.899 | 0.606 |
| Hmox1 | Mapk1 | 0 | 0 | 0 | 0 | 0 | 0 | 0.308 | 0.289 | 0.629 |
| Hmox1 | Sod1 | 0 | 0 | 0 | 0 | 0 | 0 | 0 | 0.725 | 0.722 |
| Hmox1 | Ptgs2 | 0 | 0 | 0 | 0 | 0.062 | 0 | 0 | 0.775 | 0.759 |
| Hmox1 | Jun | 0 | 0 | 0 | 0 | 0 | 0 | 0.384 | 0.601 | 0.838 |
| Hmox1 | LOC497963 | 0 | 0 | 0 | 0 | 0 | 0 | 0 | 0.647 | 0.918 |
| Hmox1 | Sqstm1 | 0 | 0 | 0 | 0 | 0.062 | 0.062 | 0 | 0.45 | 0.997 |
| Hmox1 | Th | 0 | 0 | 0 | 0 | 0.064 | 0 | 0 | 0.497 | 0.249 |
| Hmox1 | Ucp2 | 0 | 0 | 0 | 0 | 0.069 | 0 | 0 | 0.524 | 0.171 |
| Hmox1 | Sod2 | 0.085 | 0 | 0 | 0 | 0.064 | 0 | 0 | 0.691 | 0.224 |
| Hmox1 | Tp53 | 0 | 0 | 0 | 0 | 0.066 | 0 | 0 | 0.713 | 0.257 |
| Hmox1 | Mapk3 | 0 | 0 | 0 | 0 | 0 | 0 | 0.308 | 0.669 | 0.26 |
| Gsr | Sirt1 | 0.043 | 0 | 0 | 0 | 0 | 0.054 | 0 | 0.411 | 0.352 |
| Gsr | Nfe2l2 | 0 | 0 | 0 | 0 | 0.057 | 0 | 0 | 0.722 | 0.221 |
| Gsr | Sod1 | 0 | 0 | 0 | 0 | 0.084 | 0.104 | 0 | 0.833 | 0.447 |
| Gsr | Ptgs2 | 0 | 0 | 0 | 0 | 0 | 0.159 | 0 | 0.524 | 0.27 |
| Gsr | LOC497963 | 0 | 0 | 0 | 0 | 0 | 0 | 0 | 0.55 | 0.995 |
| Gsr | Hmox1 | 0 | 0 | 0 | 0 | 0.083 | 0.082 | 0 | 0.756 | 0.15 |
| Gsr | Ucp2 | 0 | 0 | 0 | 0 | 0.064 | 0.117 | 0 | 0.354 | 0.585 |
| Gsr | Mapk3 | 0 | 0 | 0 | 0 | 0.064 | 0 | 0.147 | 0.414 | 0.784 |
| Gsr | Tp53 | 0 | 0 | 0 | 0 | 0 | 0.057 | 0 | 0.559 | 0.161 |
| Gsr | Sod2 | 0.083 | 0 | 0 | 0 | 0.12 | 0.043 | 0 | 0.722 | 0.198 |
| Gsr | Gstm1 | 0 | 0 | 0 | 0 | 0.124 | 0 | 0.65 | 0.464 | 0.166 |
| Gsr | Gstp1 | 0 | 0 | 0 | 0 | 0.064 | 0 | 0.65 | 0.574 | 0.198 |
| Drd2 | Fos | 0 | 0 | 0 | 0 | 0 | 0 | 0.124 | 0.646 | 0.29 |
| Drd2 | Slc18a2 | 0 | 0 | 0 | 0 | 0.064 | 0 | 0 | 0.703 | 0.29 |
| Drd2 | Wnt5a | 0 | 0 | 0 | 0 | 0 | 0 | 0 | 0.573 | 0.296 |
| Drd2 | Prl | 0 | 0 | 0 | 0 | 0.076 | 0 | 0 | 0.652 | 0.305 |
| Drd2 | Egr1 | 0 | 0 | 0 | 0 | 0.049 | 0.046 | 0.093 | 0.377 | 0.323 |
| Drd2 | Th | 0 | 0 | 0 | 0 | 0.098 | 0 | 0 | 0.835 | 0.416 |
| Drd2 | Park2 | 0 | 0 | 0 | 0 | 0 | 0.086 | 0.211 | 0.255 | 0.224 |
| Drd2 | Lhb | 0 | 0 | 0 | 0 | 0 | 0 | 0 | 0.421 | 0.188 |
| Drd2 | Nr4a2 | 0 | 0 | 0 | 0 | 0.058 | 0.054 | 0 | 0.522 | 0.171 |
| Drd2 | Maoa | 0 | 0 | 0 | 0 | 0.044 | 0 | 0 | 0.717 | 0.162 |
| Drd2 | Slc6a3 | 0 | 0 | 0 | 0 | 0.062 | 0.425 | 0 | 0.863 | 0.336 |
| Dbh | Fos | 0 | 0 | 0 | 0 | 0 | 0 | 0 | 0.68 | 0.184 |
| Dbh | Slc18a2 | 0 | 0 | 0 | 0 | 0.06 | 0 | 0 | 0.757 | 0.181 |
| Dbh | Th | 0 | 0 | 0 | 0 | 0.105 | 0 | 0 | 0.946 | 0.236 |
| Dbh | Drd1 | 0 | 0 | 0 | 0 | 0.049 | 0 | 0 | 0.549 | 0.28 |
| Dbh | Drd2 | 0 | 0 | 0 | 0 | 0.05 | 0 | 0 | 0.646 | 0.439 |
| Dbh | Slc6a3 | 0 | 0 | 0 | 0 | 0.058 | 0 | 0 | 0.654 | 0.274 |
| Dbh | Maoa | 0 | 0 | 0 | 0 | 0 | 0 | 0.9 | 0.668 | 0.174 |
| Dlg4 | Mapk1 | 0 | 0 | 0 | 0 | 0.06 | 0.14 | 0 | 0.364 | 0.231 |
| Dlg4 | Fos | 0 | 0 | 0 | 0 | 0 | 0 | 0 | 0.583 | 0.176 |
| Dlg4 | LOC497963 | 0 | 0 | 0 | 0 | 0.064 | 0.164 | 0.501 | 0.172 | 0.241 |
| Dlg4 | Gsr | 0.043 | 0 | 0 | 0 | 0 | 0 | 0 | 0.668 | 0.226 |
| Dlg4 | Egr1 | 0 | 0 | 0 | 0 | 0 | 0.046 | 0 | 0.429 | 0.166 |
| Dlg4 | Mapk3 | 0 | 0 | 0 | 0 | 0.06 | 0.123 | 0 | 0.579 | 0.202 |
| Dlg4 | Th | 0 | 0 | 0 | 0 | 0.07 | 0 | 0 | 0.5 | 0.159 |
| Dlg4 | Drd1 | 0 | 0 | 0 | 0 | 0.134 | 0.042 | 0 | 0.656 | 0.571 |
| Dlg4 | Drd2 | 0 | 0 | 0 | 0 | 0.119 | 0.181 | 0 | 0.492 | 0.269 |
| Dlg4 | Mapk8 | 0 | 0 | 0 | 0 | 0.145 | 0 | 0 | 0.423 | 0.257 |
| Maoa | Sirt1 | 0 | 0 | 0 | 0 | 0.064 | 0.181 | 0 | 0.391 | 0.175 |
| Maoa | Slc18a2 | 0 | 0 | 0 | 0 | 0.064 | 0 | 0 | 0.757 | 0.172 |
| Maoa | Th | 0 | 0 | 0 | 0 | 0 | 0 | 0 | 0.7 | 0.21 |
| Maoa | Park2 | 0 | 0 | 0 | 0 | 0 | 0 | 0.8 | 0.235 | 0.289 |
| Maoa | Slc6a3 | 0 | 0 | 0 | 0 | 0 | 0 | 0 | 0.701 | 0.544 |
| Gpx1 | Sirt1 | 0.081 | 0 | 0 | 0 | 0 | 0.046 | 0 | 0.597 | 0.171 |
| Gpx1 | Tfam | 0 | 0 | 0 | 0 | 0 | 0 | 0 | 0.445 | 0.189 |
| Gpx1 | Nfe2l2 | 0 | 0 | 0 | 0 | 0.079 | 0 | 0 | 0.718 | 0.194 |
| Gpx1 | Sod1 | 0.045 | 0 | 0 | 0 | 0.125 | 0.237 | 0.91 | 0.846 | 0.154 |
| Gpx1 | Ptgs2 | 0 | 0 | 0 | 0 | 0 | 0 | 0 | 0.57 | 0.162 |
| Gpx1 | LOC497963 | 0 | 0 | 0 | 0 | 0 | 0 | 0 | 0.578 | 0.163 |
| Gpx1 | Hmox1 | 0 | 0 | 0 | 0 | 0.058 | 0.069 | 0 | 0.815 | 0.169 |
| Gpx1 | Gsr | 0.066 | 0 | 0 | 0 | 0.064 | 0.105 | 0.989 | 0.91 | 0.209 |
| Gpx1 | Ucp2 | 0 | 0 | 0 | 0 | 0.064 | 0 | 0 | 0.571 | 0.248 |
| Gpx1 | Gstp1 | 0 | 0 | 0 | 0 | 0.064 | 0 | 0.65 | 0.566 | 0.282 |
| Gpx1 | Sod2 | 0 | 0 | 0 | 0 | 0.059 | 0.445 | 0.91 | 0.797 | 0.298 |
| Gpx1 | Mapk3 | 0 | 0 | 0 | 0 | 0 | 0 | 0 | 0.493 | 0.328 |
| Gpx1 | Gstm1 | 0 | 0 | 0 | 0 | 0.06 | 0 | 0.65 | 0.553 | 0.386 |
| Gpx1 | Tp53 | 0 | 0 | 0 | 0 | 0.056 | 0.041 | 0 | 0.596 | 0.394 |
| Bdnf | Sirt1 | 0 | 0 | 0 | 0 | 0 | 0 | 0 | 0.619 | 0.403 |
| Bdnf | Nfe2l2 | 0 | 0 | 0 | 0 | 0 | 0 | 0 | 0.445 | 0.411 |
| Bdnf | Mapk1 | 0 | 0 | 0 | 0 | 0 | 0 | 0.17 | 0.523 | 0.751 |
| Bdnf | Sod1 | 0 | 0 | 0 | 0 | 0 | 0 | 0 | 0.531 | 0.942 |
| Bdnf | Ptgs2 | 0 | 0 | 0 | 0 | 0.058 | 0 | 0 | 0.619 | 0.999 |
| Bdnf | Fos | 0 | 0 | 0 | 0 | 0 | 0 | 0 | 0.829 | 0.239 |
| Bdnf | Cat | 0 | 0 | 0 | 0 | 0 | 0 | 0 | 0.579 | 0.18 |
| Bdnf | Jun | 0 | 0 | 0 | 0 | 0 | 0 | 0 | 0.547 | 0.209 |
| Bdnf | Slc18a2 | 0 | 0 | 0 | 0 | 0 | 0 | 0 | 0.663 | 0.157 |
| Bdnf | Casp3 | 0 | 0 | 0 | 0 | 0 | 0 | 0.297 | 0.757 | 0.187 |
| Bdnf | Casp9 | 0 | 0 | 0 | 0 | 0 | 0 | 0.297 | 0.498 | 0.198 |
| Bdnf | Creb1 | 0 | 0 | 0 | 0 | 0 | 0 | 0 | 0.901 | 0.226 |
| Bdnf | Hmox1 | 0 | 0 | 0 | 0 | 0 | 0 | 0 | 0.625 | 0.232 |
| Bdnf | Gsr | 0 | 0 | 0 | 0 | 0 | 0 | 0 | 0.445 | 0.27 |
| Bdnf | Prl | 0 | 0 | 0 | 0 | 0 | 0 | 0 | 0.432 | 0.192 |
| Bdnf | Egr1 | 0 | 0 | 0 | 0 | 0 | 0.044 | 0.163 | 0.726 | 0.696 |
| Bdnf | Mapk3 | 0 | 0 | 0 | 0 | 0 | 0 | 0.17 | 0.72 | 0.372 |
| Bdnf | Th | 0 | 0 | 0 | 0 | 0.057 | 0 | 0 | 0.803 | 0.2 |
| Bdnf | Drd1 | 0 | 0 | 0 | 0 | 0.076 | 0 | 0 | 0.606 | 0.157 |
| Bdnf | Drd2 | 0 | 0 | 0 | 0 | 0.057 | 0 | 0 | 0.722 | 0.348 |
| Bdnf | Nr4a2 | 0 | 0 | 0 | 0 | 0.08 | 0 | 0 | 0.621 | 0.241 |
| Bdnf | Slc6a3 | 0 | 0 | 0 | 0 | 0.057 | 0 | 0 | 0.589 | 0.187 |
| Bdnf | Tp53 | 0 | 0 | 0 | 0 | 0 | 0 | 0.588 | 0.578 | 0.215 |
| Bdnf | Dbh | 0 | 0 | 0 | 0 | 0 | 0 | 0 | 0.498 | 0.297 |
| Bdnf | Dlg4 | 0 | 0 | 0 | 0 | 0.078 | 0 | 0 | 0.875 | 0.175 |
| Bdnf | Mapk8 | 0 | 0 | 0 | 0 | 0.065 | 0 | 0.629 | 0.297 | 0.156 |
| Bdnf | Maoa | 0 | 0 | 0 | 0 | 0.058 | 0 | 0 | 0.67 | 0.157 |
| Adora2a | Mapk8 | 0 | 0 | 0 | 0 | 0 | 0.054 | 0.148 | 0.318 | 0.161 |
| Adora2a | Slc6a3 | 0 | 0 | 0 | 0 | 0 | 0.044 | 0 | 0.405 | 0.176 |
| Adora2a | Mapk3 | 0 | 0 | 0 | 0 | 0 | 0 | 0.124 | 0.367 | 0.186 |
| Adora2a | Th | 0 | 0 | 0 | 0 | 0 | 0 | 0 | 0.477 | 0.304 |
| Adora2a | Drd1 | 0 | 0 | 0 | 0.667 | 0.318 | 0 | 0 | 0.785 | 0.408 |
| Adora2a | Creb1 | 0 | 0 | 0 | 0 | 0 | 0.054 | 0 | 0.546 | 0.957 |
| Adora2a | Bdnf | 0 | 0 | 0 | 0 | 0 | 0 | 0 | 0.572 | 0.206 |
| Adora2a | Fshb | 0 | 0 | 0 | 0 | 0 | 0 | 0.6 | 0.042 | 0.198 |
| Adora2a | Cga | 0 | 0 | 0 | 0 | 0 | 0 | 0.6 | 0 | 0.15 |
| Adora2a | Fos | 0 | 0 | 0 | 0 | 0 | 0 | 0.124 | 0.582 | 0.155 |
| Adora2a | Drd2 | 0 | 0 | 0 | 0.657 | 0.123 | 0.621 | 0 | 0.982 | 0.155 |
| Mapk8 | Sirt1 | 0 | 0 | 0 | 0 | 0.064 | 0.192 | 0 | 0.36 | 0.167 |
| Mapk8 | Wnt5a | 0 | 0 | 0 | 0 | 0.05 | 0 | 0.629 | 0.16 | 0.195 |
| Mapk8 | Nfkb1 | 0 | 0 | 0 | 0 | 0.049 | 0.104 | 0.8 | 0.538 | 0.218 |
| Mapk8 | Park2 | 0 | 0 | 0 | 0 | 0.059 | 0.317 | 0 | 0.207 | 0.235 |
| Mapk8 | Rela | 0 | 0 | 0 | 0 | 0 | 0.053 | 0.8 | 0.316 | 0.315 |
| Mapk8 | Tp53 | 0 | 0 | 0 | 0 | 0 | 0.521 | 0.897 | 0.552 | 0.343 |
| Slc6a3 | Th | 0 | 0 | 0 | 0 | 0.097 | 0 | 0 | 0.79 | 0.266 |
| Mapk3 | Sirt1 | 0 | 0 | 0 | 0 | 0.049 | 0.041 | 0 | 0.62 | 0.203 |
| Mapk3 | Nfe2l2 | 0 | 0 | 0 | 0 | 0.05 | 0.134 | 0 | 0.567 | 0.205 |
| Mapk3 | Sod1 | 0 | 0 | 0 | 0 | 0.049 | 0.284 | 0 | 0.471 | 0.205 |
| Mapk3 | Ptgs2 | 0 | 0 | 0 | 0 | 0 | 0.416 | 0 | 0.699 | 0.208 |
| Mapk3 | Prl | 0 | 0 | 0 | 0 | 0 | 0 | 0 | 0.5 | 0.332 |
| Mapk3 | Sod2 | 0 | 0 | 0 | 0 | 0.056 | 0.089 | 0 | 0.45 | 0.951 |
| Mapk3 | Sqstm1 | 0 | 0 | 0 | 0 | 0 | 0.404 | 0 | 0.562 | 0.202 |
| Mapk3 | Th | 0 | 0 | 0 | 0 | 0 | 0.34 | 0.342 | 0.506 | 0.227 |
| Mapk3 | Nfkb1 | 0 | 0 | 0 | 0 | 0 | 0.127 | 0.615 | 0.378 | 0.205 |
| Mapk3 | Mapk8 | 0 | 0 |  | 0.882 | 0.079 | 0.09 | 0.735 | 0.653 | 0.284 |
| Mapk3 | Rela | 0 | 0 | 0 | 0 | 0 | 0.046 | 0.615 | 0.476 | 0.203 |
| Mapk3 | Prkaca | 0 | 0 |  | 0.611 | 0.064 | 0.169 | 0.913 | 0.197 | 0.199 |
| Mapk3 | Tp53 | 0 | 0 | 0 | 0 | 0.08 | 0.452 | 0.845 | 0.777 | 0.209 |
| Drd1 | Fshb | 0 | 0 | 0 | 0 | 0 | 0 | 0.6 | 0.062 | 0.15 |
| Drd1 | Fos | 0 | 0 | 0 | 0 | 0 | 0 | 0.124 | 0.675 | 0.998 |
| Drd1 | Slc18a2 | 0 | 0 | 0 | 0 | 0 | 0 | 0 | 0.645 | 0.175 |
| Drd1 | Egr1 | 0 | 0 | 0 | 0 | 0 | 0.046 | 0.093 | 0.422 | 0.158 |
| Drd1 | Th | 0 | 0 | 0 | 0 | 0.064 | 0 | 0 | 0.799 | 0.327 |
| Drd1 | Maoa | 0 | 0 | 0 | 0 | 0.061 | 0 | 0 | 0.534 | 0.189 |
| Drd1 | Drd2 | 0 | 0 | 0 | 0.761 | 0.261 | 0.413 | 0 | 0.943 | 0.946 |
| Drd1 | Slc6a3 | 0 | 0 | 0 | 0 | 0.054 | 0.044 | 0 | 0.646 | 0.166 |
| Fshb | Gh1 | 0 | 0 | 0 | 0 | 0.586 | 0 | 0 | 0.265 | 0.152 |
| Fshb | Prl | 0 | 0 | 0 | 0 | 0.35 | 0 | 0 | 0.735 | 0.208 |
| Cga | Fshb | 0 | 0 | 0 | 0 | 0.573 | 0.939 | 0.8 | 0.815 | 0.165 |
| Cga | Drd1 | 0 | 0 | 0 | 0 | 0 | 0 | 0.6 | 0.127 | 0.508 |
| Cga | Gh1 | 0 | 0 | 0 | 0 | 0.701 | 0 | 0 | 0.426 | 0.156 |
| Cga | Prl | 0 | 0 | 0 | 0 | 0.341 | 0 | 0 | 0.879 | 0.248 |
| Cga | Lhb | 0 | 0 | 0 | 0 | 0.179 | 0.545 | 0.8 | 0.839 | 0.178 |
| Arg1 | Ptgs2 | 0 | 0 | 0 | 0 | 0.046 | 0 | 0 | 0.542 | 0.189 |
| Arg1 | Cat | 0 | 0 | 0 | 0 | 0.068 | 0.054 | 0 | 0.417 | 0.198 |
| Arg1 | LOC497963 | 0 | 0 | 0 | 0 | 0.056 | 0 | 0.9 | 0.787 | 0.263 |
| Arg1 | Hmox1 | 0 | 0 | 0 | 0 | 0 | 0 | 0 | 0.454 | 0.891 |
| LOC497963 | Nfe2l2 | 0 | 0 | 0 | 0 | 0 | 0 | 0.321 | 0.504 | 0.183 |
| LOC497963 | Sod1 | 0 | 0 | 0 | 0 | 0 | 0.046 | 0 | 0.434 | 0.24 |
| LOC497963 | Ptgs2 | 0 | 0 | 0 | 0 | 0.064 | 0.16 | 0 | 0.709 | 0.365 |
| LOC497963 | Mapk3 | 0 | 0 | 0 | 0 | 0.048 | 0 | 0 | 0.404 | 0.205 |
| LOC497963 | Prkaca | 0 | 0 | 0 | 0 | 0 | 0.133 | 0.385 | 0 | 0.256 |
| LOC497963 | Rela | 0 | 0 | 0 | 0 | 0 | 0.528 | 0.55 | 0.298 | 0.256 |
| Atp1a1 | Mapk1 | 0 | 0 | 0 | 0 | 0.121 | 0.114 | 0.6 | 0.083 | 0.458 |
| Atp1a1 | Mapk3 | 0 | 0 | 0 | 0 | 0.231 | 0.099 | 0.6 | 0.099 | 0.224 |
| Atp1a1 | Prkaca | 0 | 0 | 0 | 0 | 0.064 | 0.056 | 0.6 | 0.056 | 0.188 |
| Mapk1 | Th | 0 | 0 | 0 | 0 | 0 | 0.204 | 0.342 | 0.228 | 0.169 |
| Mapk1 | Ptgs2 | 0 | 0 | 0 | 0 | 0 | 0.415 | 0 | 0.398 | 0.174 |
| Mapk1 | Sqstm1 | 0 | 0 | 0 | 0 | 0 | 0.516 | 0 | 0.395 | 0.545 |
| Mapk1 | Rela | 0 | 0 | 0 | 0 | 0 | 0.054 | 0.637 | 0.3 | 0.158 |
| Mapk1 | Nfkb1 | 0 | 0 | 0 | 0 | 0.047 | 0.127 | 0.629 | 0.362 | 0.214 |
| Mapk1 | Mapk8 | 0 | 0 |  | 0.892 | 0.079 | 0.09 | 0.735 | 0.639 | 0.176 |
| Mapk1 | Prkaca | 0 | 0 |  | 0.611 | 0.064 | 0.169 | 0.924 | 0.258 | 0.384 |
| Mapk1 | Tp53 | 0 | 0 | 0 | 0 | 0 | 0.666 | 0.845 | 0.605 | 0.253 |
| Mapk1 | Mapk3 | 0 | 0 |  | 0.983 | 0 | 0.827 | 0.919 | 0.845 | 0.198 |
| Prkaca | Th | 0 | 0 | 0 | 0 | 0.056 | 0.504 | 0 | 0.046 | 0.34 |
| Prkaca | Rela | 0 | 0 | 0 | 0 | 0.05 | 0.522 | 0.959 | 0.049 | 0.388 |
| Bax | Sirt1 | 0 | 0 | 0 | 0 | 0 | 0 | 0.853 | 0.448 | 0.365 |
| Bax | Nfe2l2 | 0 | 0 | 0 | 0 | 0 | 0 | 0 | 0.523 | 0.263 |
| Bax | Ptgs2 | 0 | 0 | 0 | 0 | 0 | 0 | 0 | 0.52 | 0.26 |
| Bax | Bcl2 | 0 | 0 | 0 | 0.699 | 0 | 0.813 | 0.8 | 0.847 | 0.164 |
| Bax | Cat | 0 | 0 | 0 | 0 | 0 | 0 | 0 | 0.571 | 0.164 |
| Bax | Casp3 | 0 | 0 | 0 | 0 | 0 | 0.286 | 0 | 0.86 | 0.209 |
| Bax | Casp9 | 0 | 0 | 0 | 0 | 0 | 0.286 | 0 | 0.743 | 0.252 |
| Bax | Creb1 | 0 | 0 | 0 | 0 | 0 | 0 | 0.288 | 0.358 | 0.153 |
| Bax | Hmox1 | 0 | 0 | 0 | 0 | 0.061 | 0 | 0 | 0.57 | 0.288 |
| Bax | Bcl2l1 | 0 | 0 | 0 | 0 | 0.05 | 0.598 | 0 | 0.749 | 0.175 |
| Bax | Mapk3 | 0 | 0 | 0 | 0 | 0 | 0.062 | 0.219 | 0.325 | 0.194 |
| Bax | Becn1 | 0 | 0 | 0 | 0 | 0 | 0.421 | 0 | 0.499 | 0.272 |
| Bax | Rela | 0 | 0 | 0 | 0 | 0 | 0.058 | 0.341 | 0.118 | 0.56 |
| Bax | Bdnf | 0 | 0 | 0 | 0 | 0 | 0 | 0 | 0.447 | 0.16 |
| Bax | Gpx1 | 0 | 0 | 0 | 0 | 0 | 0 | 0 | 0.449 | 0.321 |
| Bax | Mapk8 | 0 | 0 | 0 | 0 | 0 | 0.137 | 0.307 | 0.197 | 0.268 |
| Bax | Cycs | 0 | 0 | 0 | 0 | 0 | 0.135 | 0.312 | 0.398 | 0.235 |
| Bax | Park2 | 0 | 0 | 0 | 0 | 0 | 0.193 | 0.8 | 0.188 | 0.312 |
| Bax | Prkaca | 0 | 0 | 0 | 0 | 0 | 0.046 | 0.857 | 0 | 0.574 |
| Bax | Tp53 | 0 | 0 | 0 | 0 | 0.098 | 0.217 | 0.859 | 0.724 | 0.18 |
| Sirt1 | Sod1 | 0.045 | 0 | 0 | 0 | 0 | 0 | 0 | 0.627 | 0.183 |
| Sirt1 | Ucp2 | 0 | 0 | 0 | 0 | 0.064 | 0.083 | 0 | 0.701 | 0.189 |
| Sirt1 | Sod2 | 0 | 0 | 0 | 0 | 0.064 | 0.058 | 0 | 0.799 | 0.2 |
| Sirt1 | Tfam | 0 | 0 | 0 | 0 | 0.06 | 0.102 | 0 | 0.85 | 0.209 |
| Sirt1 | Tp53 | 0 | 0 | 0 | 0 | 0.056 | 0.696 | 0.914 | 0.96 | 0.253 |
| Nfe2l2 | Sirt1 | 0 | 0 | 0 | 0 | 0 | 0.043 | 0 | 0.761 | 0.272 |
| Nfe2l2 | Tfam | 0 | 0 | 0 | 0 | 0 | 0.054 | 0 | 0.502 | 0.282 |
| Nfe2l2 | Sqstm1 | 0 | 0 | 0 | 0 | 0.056 | 0.088 | 0 | 0.538 | 0.557 |
| Nfe2l2 | Ptgs2 | 0 | 0 | 0 | 0 | 0 | 0 | 0 | 0.629 | 0.589 |
| Nfe2l2 | Sod2 | 0 | 0 | 0 | 0 | 0 | 0 | 0 | 0.636 | 0.951 |
| Nfe2l2 | Sod1 | 0 | 0 | 0 | 0 | 0 | 0 | 0 | 0.667 | 0.98 |
| Nfe2l2 | Tp53 | 0 | 0 | 0 | 0 | 0 | 0.164 | 0 | 0.673 | 0.995 |
| Bcl2 | Mapk1 | 0 | 0 | 0 | 0 | 0 | 0.203 | 0.924 | 0.255 | 0.998 |
| Bcl2 | Sod1 | 0 | 0 | 0 | 0 | 0 | 0.125 | 0.8 | 0.096 | 0.226 |
| Bcl2 | Wnt5a | 0 | 0 | 0 | 0 | 0 | 0 | 0 | 0.403 | 0.193 |
| Bcl2 | Rela | 0 | 0 | 0 | 0 | 0.047 | 0.114 | 0.341 | 0.093 | 0.186 |
| Bcl2 | Casp9 | 0 | 0 | 0 | 0 | 0 | 0.309 | 0 | 0.486 | 0.219 |
| Bcl2 | Creb1 | 0 | 0 | 0 | 0 | 0 | 0 | 0.584 | 0.154 | 0.235 |
| Bcl2 | Prkaca | 0 | 0 | 0 | 0 | 0 | 0.077 | 0.637 | 0.044 | 0.277 |
| Bcl2 | Cycs | 0 | 0 | 0 | 0 | 0 | 0.193 | 0.512 | 0.278 | 0.152 |
| Bcl2 | Casp3 | 0 | 0 | 0 | 0 | 0 | 0.286 | 0 | 0.609 | 0.259 |
| Bcl2 | Bcl2l1 | 0 | 0 | 0 | 0.716 | 0 | 0.707 | 0.615 | 0.425 | 0.152 |
| Bcl2 | Mapk8 | 0 | 0 | 0 | 0 | 0 | 0.216 | 0.905 | 0.162 | 0.152 |
| Bcl2 | Mapk3 | 0 | 0 | 0 | 0 | 0 | 0.159 | 0.913 | 0.182 | 0.153 |
| Bcl2 | Tp53 | 0 | 0 | 0 | 0 | 0 | 0.696 | 0.914 | 0.526 | 0.159 |
| Bcl2 | Becn1 | 0 | 0 | 0 | 0 | 0 | 0.971 | 0.8 | 0.799 | 0.16 |
| Casp9 | Sirt1 | 0 | 0 | 0 | 0 | 0.051 | 0 | 0 | 0.523 | 0.165 |
| Casp9 | Nfe2l2 | 0 | 0 | 0 | 0 | 0 | 0.065 | 0 | 0.477 | 0.172 |
| Casp9 | Mapk1 | 0 | 0 | 0 | 0 | 0 | 0.12 | 0.916 | 0.429 | 0.197 |
| Casp9 | Sod1 | 0 | 0 | 0 | 0 | 0 | 0.092 | 0 | 0.439 | 0.203 |
| Casp9 | Ptgs2 | 0 | 0 | 0 | 0 | 0 | 0 | 0 | 0.619 | 0.298 |
| Casp9 | Fos | 0 | 0 | 0 | 0 | 0 | 0 | 0 | 0.425 | 0.996 |
| Casp9 | Cat | 0 | 0 | 0 | 0 | 0 | 0 | 0 | 0.68 | 0.185 |
| Casp9 | Jun | 0 | 0 | 0 | 0 | 0 | 0.193 | 0 | 0.566 | 0.201 |
| Casp9 | Sod2 | 0 | 0 | 0 | 0 | 0.051 | 0 | 0 | 0.432 | 0.275 |
| Casp9 | Gsr | 0 | 0 | 0 | 0 | 0 | 0 | 0 | 0.445 | 0.365 |
| Casp9 | Sqstm1 | 0 | 0 | 0 | 0 | 0 | 0.143 | 0 | 0.396 | 0.179 |
| Casp9 | Creb1 | 0 | 0 | 0 | 0 | 0 | 0 | 0 | 0.522 | 0.305 |
| Casp9 | Rela | 0 | 0 | 0 | 0 | 0 | 0 | 0.324 | 0.356 | 0.232 |
| Casp9 | Gpx1 | 0 | 0 | 0 | 0 | 0 | 0 | 0 | 0.571 | 0.234 |
| Casp9 | Hmox1 | 0 | 0 | 0 | 0 | 0 | 0 | 0 | 0.627 | 0.151 |
| Casp9 | Cycs | 0 | 0 | 0 | 0 | 0.064 | 0.322 | 0.512 | 0.536 | 0.172 |
| Casp9 | Tp53 | 0 | 0 | 0 | 0 | 0.058 | 0.085 | 0 | 0.88 | 0.186 |
| Casp9 | Mapk3 | 0 | 0 | 0 | 0 | 0 | 0.12 | 0.916 | 0.63 | 0.204 |
| Bcl2l1 | Sirt1 | 0 | 0 | 0 | 0 | 0.05 | 0 | 0.296 | 0.53 | 0.463 |
| Bcl2l1 | Nfe2l2 | 0 | 0 | 0 | 0 | 0 | 0 | 0 | 0.445 | 0.167 |
| Bcl2l1 | Mapk1 | 0 | 0 | 0 | 0 | 0 | 0.127 | 0.615 | 0.345 | 0.167 |
| Bcl2l1 | Ptgs2 | 0 | 0 | 0 | 0 | 0 | 0 | 0 | 0.636 | 0.234 |
| Bcl2l1 | Fos | 0 | 0 | 0 | 0 | 0.058 | 0 | 0 | 0.538 | 0.269 |
| Bcl2l1 | Cat | 0 | 0 | 0 | 0 | 0 | 0 | 0 | 0.608 | 0.155 |
| Bcl2l1 | Jun | 0 | 0 | 0 | 0 | 0.06 | 0 | 0 | 0.598 | 0.189 |
| Bcl2l1 | Casp3 | 0 | 0 | 0 | 0 | 0 | 0.286 | 0 | 0.915 | 0.211 |
| Bcl2l1 | Casp9 | 0 | 0 | 0 | 0 | 0.05 | 0.424 | 0 | 0.905 | 0.276 |
| Bcl2l1 | Creb1 | 0 | 0 | 0 | 0 | 0 | 0 | 0.501 | 0.571 | 0.327 |
| Bcl2l1 | Hmox1 | 0 | 0 | 0 | 0 | 0 | 0 | 0 | 0.668 | 0.257 |
| Bcl2l1 | Sqstm1 | 0 | 0 | 0 | 0 | 0.064 | 0.159 | 0 | 0.309 | 0.206 |
| Bcl2l1 | Park2 | 0 | 0 | 0 | 0 | 0 | 0.193 | 0 | 0.302 | 0.27 |
| Bcl2l1 | Egr1 | 0 | 0 | 0 | 0 | 0 | 0.043 | 0.136 | 0.367 | 0.197 |
| Bcl2l1 | Nfkb1 | 0 | 0 | 0 | 0 | 0 | 0.054 | 0.148 | 0.367 | 0.257 |
| Bcl2l1 | Nr4a2 | 0 | 0 | 0 | 0 | 0 | 0.132 | 0.257 | 0.233 | 0.237 |
| Bcl2l1 | Sod2 | 0 | 0 | 0 | 0 | 0 | 0 | 0 | 0.525 | 0.206 |
| Bcl2l1 | Ccnd2 | 0 | 0 | 0 | 0 | 0.048 | 0 | 0 | 0.538 | 0.58 |
| Bcl2l1 | Bdnf | 0 | 0 | 0 | 0 | 0 | 0 | 0 | 0.544 | 0.224 |
| Bcl2l1 | Prkaca | 0 | 0 | 0 | 0 | 0.048 | 0.056 | 0.584 | 0.065 | 0.17 |
| Bcl2l1 | Mapk8 | 0 | 0 | 0 | 0 | 0 | 0.317 | 0.442 | 0.338 | 0.179 |
| Bcl2l1 | Rela | 0 | 0 | 0 | 0 | 0.057 | 0.099 | 0.341 | 0.585 | 0.326 |
| Bcl2l1 | Cycs | 0 | 0 | 0 | 0 | 0 | 0.31 | 0.508 | 0.376 | 0.226 |
| Bcl2l1 | Mapk3 | 0 | 0 | 0 | 0 | 0.05 | 0.127 | 0.57 | 0.711 | 0.201 |
| Bcl2l1 | Tp53 | 0 | 0 | 0 | 0 | 0 | 0.711 | 0.508 | 0.993 | 0.173 |
| Bcl2l1 | Becn1 | 0 | 0 | 0 | 0 | 0 | 0.806 | 0 | 0.995 | 0.173 |
| Becn1 | Sirt1 | 0 | 0 | 0 | 0 | 0.048 | 0 | 0 | 0.738 | 0.201 |
| Becn1 | Tfam | 0 | 0 | 0 | 0 | 0 | 0.098 | 0 | 0.473 | 0.25 |
| Becn1 | Nfe2l2 | 0 | 0 | 0 | 0 | 0 | 0 | 0 | 0.471 | 0.28 |
| Becn1 | Sod1 | 0 | 0 | 0 | 0 | 0.064 | 0 | 0 | 0.77 | 0.331 |
| Becn1 | Cat | 0 | 0 | 0 | 0 | 0 | 0 | 0 | 0.565 | 0.17 |
| Becn1 | Jun | 0 | 0 | 0 | 0 | 0 | 0 | 0 | 0.517 | 0.152 |
| Becn1 | Casp3 | 0 | 0 | 0 | 0 | 0 | 0.117 | 0 | 0.847 | 0.16 |
| Becn1 | Casp9 | 0 | 0 | 0 | 0 | 0 | 0.117 | 0 | 0.771 | 0.172 |
| Becn1 | Hmox1 | 0 | 0 | 0 | 0 | 0 | 0 | 0 | 0.548 | 0.178 |
| Becn1 | Sod2 | 0 | 0 | 0 | 0 | 0 | 0 | 0 | 0.523 | 0.18 |
| Becn1 | Mapk3 | 0 | 0 | 0 | 0 | 0.05 | 0 | 0 | 0.686 | 0.205 |
| Becn1 | Tp53 | 0 | 0 | 0 | 0 | 0 | 0.092 | 0 | 0.798 | 0.231 |
| Becn1 | Sqstm1 | 0 | 0 | 0 | 0 | 0 | 0.114 | 0 | 0.887 | 0.243 |
| Becn1 | Park2 | 0 | 0 | 0 | 0 | 0.085 | 0.826 | 0 | 0.936 | 0.386 |
| Rela | Sirt1 | 0 | 0 | 0 | 0 | 0 | 0.524 | 0.8 | 0.937 | 0.506 |
| Rela | Tfam | 0 | 0 | 0 | 0 | 0 | 0.054 | 0.325 | 0.202 | 0.938 |
| Rela | Sod2 | 0 | 0 | 0 | 0 | 0 | 0 | 0.637 | 0.393 | 0.998 |
| Rela | Tp53 | 0 | 0 | 0 | 0 | 0.076 | 0.163 | 0 | 0.9 | 0.196 |
| Rela | Sqstm1 | 0 | 0 | 0 | 0 | 0.058 | 0.054 | 0.961 | 0.23 | 0.56 |
| Cycs | Tfam | 0 | 0 | 0 | 0 | 0.064 | 0 | 0 | 0.489 | 0.178 |
| Cycs | Gsr | 0 | 0 | 0 | 0 | 0.075 | 0.255 | 0 | 0.224 | 0.203 |
| Cycs | Sod2 | 0 | 0 | 0 | 0 | 0.107 | 0 | 0 | 0.461 | 0.912 |
| Park2 | Tfam | 0 | 0 | 0 | 0 | 0.056 | 0 | 0 | 0.714 | 0.298 |
| Park2 | Sod1 | 0 | 0 | 0 | 0 | 0 | 0.139 | 0 | 0.535 | 0.256 |
| Park2 | Slc18a2 | 0 | 0 | 0 | 0 | 0 | 0 | 0 | 0.474 | 0.179 |
| Park2 | Sod2 | 0 | 0 | 0 | 0 | 0 | 0.355 | 0 | 0.368 | 0.183 |
| Park2 | Th | 0 | 0 | 0 | 0 | 0 | 0 | 0 | 0.714 | 0.401 |
| Park2 | Tp53 | 0 | 0 | 0 | 0 | 0 | 0.164 | 0 | 0.398 | 0.151 |
| Park2 | Slc6a3 | 0 | 0 | 0 | 0 | 0 | 0.419 | 0 | 0.51 | 0.664 |
| Park2 | Sqstm1 | 0 | 0 | 0 | 0 | 0 | 0.648 | 0 | 0.638 | 0.195 |
| Tp53 | Ucp2 | 0 | 0 | 0 | 0 | 0.104 | 0 | 0 | 0.587 | 0.156 |
| Sod1 | Tfam | 0 | 0 | 0 | 0 | 0.064 | 0 | 0 | 0.518 | 0.154 |
| Sod1 | Th | 0 | 0 | 0 | 0 | 0 | 0 | 0 | 0.419 | 0.159 |
| Sod1 | Ucp2 | 0 | 0 | 0 | 0 | 0 | 0 | 0 | 0.471 | 0.207 |
| Sod1 | Tp53 | 0 | 0 | 0 | 0 | 0 | 0 | 0 | 0.55 | 0.177 |
| Sod1 | Sqstm1 | 0 | 0 | 0 | 0 | 0.099 | 0.498 | 0 | 0.636 | 0.156 |
| Sod1 | Sod2 | 0.043 | 0 | 0 | 0 | 0.203 | 0.924 | 0.924 | 0.886 | 0.17 |
| Jun | Sirt1 | 0 | 0 | 0 | 0 | 0 | 0.679 | 0 | 0.687 | 0.358 |
| Jun | Tfam | 0 | 0 | 0 | 0 | 0 | 0.12 | 0.244 | 0.191 | 0.168 |
| Jun | Nfe2l2 | 0 | 0 | 0 | 0 | 0 | 0.164 | 0.358 | 0.651 | 0.158 |
| Jun | Mapk1 | 0 | 0 | 0 | 0 | 0 | 0.697 | 0.924 | 0.589 | 0.264 |
| Jun | Ptgs2 | 0 | 0 | 0 | 0 | 0.058 | 0 | 0 | 0.671 | 0.32 |
| Jun | Sod2 | 0 | 0 | 0 | 0 | 0 | 0 | 0 | 0.416 | 0.39 |
| Jun | Nr4a2 | 0 | 0 | 0 | 0 | 0.064 | 0.1 | 0 | 0.442 | 0.151 |
| Jun | Nfkb1 | 0 | 0 | 0 | 0 | 0.064 | 0.044 | 0.206 | 0.604 | 0.96 |
| Jun | LOC497963 | 0 | 0 | 0 | 0 | 0 | 0 | 0.55 | 0.457 | 0.172 |
| Jun | Tp53 | 0 | 0 | 0 | 0 | 0 | 0.054 | 0 | 0.818 | 0.311 |
| Jun | Prkaca | 0 | 0 | 0 | 0 | 0 | 0 | 0.8 | 0.168 | 0.283 |
| Jun | Rela | 0 | 0 | 0 | 0 | 0.058 | 0.311 | 0.637 | 0.754 | 0.163 |
| Jun | Mapk3 | 0 | 0 | 0 | 0 | 0 | 0.664 | 0.919 | 0.733 | 0.237 |
| Jun | Mapk8 | 0 | 0 | 0 | 0 | 0 | 0.886 | 0.962 | 0.786 | 0.153 |
| Sqstm1 | Tp53 | 0 | 0 | 0 | 0 | 0 | 0.331 | 0 | 0.439 | 0.157 |
| Egr1 | Mapk1 | 0 | 0 | 0 | 0 | 0 | 0.132 | 0.924 | 0.424 | 0.161 |
| Egr1 | Ptgs2 | 0 | 0 | 0 | 0 | 0.068 | 0.043 | 0 | 0.534 | 0.183 |
| Egr1 | Fshb | 0 | 0 | 0 | 0 | 0.057 | 0 | 0 | 0.52 | 0.188 |
| Egr1 | Fos | 0 | 0 | 0 | 0 | 0.945 | 0.08 | 0.134 | 0.946 | 0.309 |
| Egr1 | Jun | 0 | 0 | 0 | 0 | 0.525 | 0.104 | 0.153 | 0.813 | 0.154 |
| Egr1 | Hmox1 | 0 | 0 | 0 | 0 | 0 | 0 | 0 | 0.449 | 0.637 |
| Egr1 | Th | 0 | 0 | 0 | 0 | 0 | 0 | 0 | 0.445 | 0.196 |
| Egr1 | Nfkb1 | 0 | 0 | 0 | 0 | 0.057 | 0.101 | 0.126 | 0.347 | 0.793 |
| Egr1 | Lhb | 0 | 0 | 0 | 0 | 0 | 0 | 0 | 0.48 | 0.177 |
| Egr1 | Rela | 0 | 0 | 0 | 0 | 0 | 0.114 | 0.208 | 0.357 | 0.181 |
| Egr1 | Nr4a2 | 0 | 0 | 0 | 0 | 0.094 | 0.078 | 0 | 0.591 | 0.171 |
| Egr1 | Tp53 | 0 | 0 | 0 | 0 | 0 | 0.536 | 0.194 | 0.762 | 0.174 |
| Egr1 | Mapk3 | 0 | 0 | 0 | 0 | 0 | 0.132 | 0.913 | 0.664 | 0.186 |
| Nfkb1 | Sirt1 | 0 | 0 | 0 | 0 | 0.06 | 0.217 | 0.8 | 0.249 | 0.334 |
| Nfkb1 | Ptgs2 | 0 | 0 | 0 | 0 | 0.064 | 0.07 | 0 | 0.475 | 0.198 |
| Nfkb1 | Tp53 | 0 | 0 | 0 | 0 | 0 | 0.137 | 0.232 | 0.541 | 0.161 |
| Nfkb1 | Prkaca | 0 | 0 | 0 | 0 | 0.115 | 0.077 | 0.959 | 0.126 | 0.2 |
| Nfkb1 | Sqstm1 | 0 | 0 | 0 | 0 | 0.063 | 0 | 0.961 | 0.221 | 0.24 |
| Nfkb1 | Rela | 0 | 0 | 0 | 0.775 | 0.064 | 0.992 | 0.924 | 0.957 | 0.224 |
| Nr4a2 | Slc18a2 | 0 | 0 | 0 | 0 | 0 | 0 | 0 | 0.763 | 0.504 |
| Nr4a2 | Th | 0 | 0 | 0 | 0 | 0 | 0 | 0 | 0.83 | 0.305 |
| Nr4a2 | Slc6a3 | 0 | 0 | 0 | 0 | 0 | 0 | 0 | 0.54 | 0.483 |
| Nr4a2 | Tp53 | 0 | 0 | 0 | 0 | 0 | 0.133 | 0 | 0.539 | 0.156 |
| Sod2 | Tfam | 0 | 0 | 0 | 0 | 0.064 | 0 | 0 | 0.705 | 0.269 |
| Sod2 | Ucp2 | 0 | 0 | 0 | 0 | 0.064 | 0 | 0 | 0.622 | 0.172 |
| Sod2 | Tp53 | 0 | 0 | 0 | 0 | 0 | 0.16 | 0 | 0.718 | 0.641 |
| Ccnd2 | Fos | 0 | 0 | 0 | 0 | 0 | 0.082 | 0 | 0.426 | 0.239 |
| Ccnd2 | Jun | 0 | 0 | 0 | 0 | 0 | 0.06 | 0 | 0.423 | 0.43 |
| Ccnd2 | Mapk3 | 0 | 0 | 0 | 0 | 0.063 | 0.054 | 0.206 | 0.443 | 0.667 |
| Ccnd2 | Rela | 0 | 0 | 0 | 0 | 0.049 | 0.146 | 0.501 | 0.19 | 0.171 |
| Ccnd2 | Tp53 | 0 | 0 | 0 | 0 | 0 | 0.068 | 0 | 0.648 | 0.202 |
| Ccnd2 | Cdkn1c | 0 | 0 | 0 | 0 | 0.061 | 0.732 | 0.735 | 0.598 | 0.183 |
| Prl | Tp53 | 0 | 0 | 0 | 0 | 0 | 0 | 0 | 0.46 | 0.161 |
| Prl | Th | 0 | 0 | 0 | 0 | 0 | 0 | 0 | 0.684 | 0.52 |
| Tfam | Ucp2 | 0 | 0 | 0 | 0 | 0 | 0 | 0 | 0.702 | 0.799 |
| Tfam | Tp53 | 0 | 0 | 0 | 0 | 0 | 0.164 | 0 | 0.982 | 0.238 |
| Cyp2e1 | Sirt1 | 0 | 0 | 0 | 0 | 0 | 0 | 0 | 0.425 | 0.158 |
| Cyp2e1 | Nfe2l2 | 0 | 0 | 0 | 0 | 0 | 0 | 0 | 0.581 | 0.581 |
| Cyp2e1 | Ptgs2 | 0 | 0 | 0 | 0 | 0.056 | 0.042 | 0.905 | 0.463 | 0.947 |
| Cyp2e1 | Jun | 0 | 0 | 0 | 0 | 0 | 0.413 | 0 | 0.357 | 0.606 |
| Cyp2e1 | LOC497963 | 0.043 | 0 | 0 | 0 | 0 | 0.082 | 0.104 | 0.354 | 0.423 |
| Cyp2e1 | Gsr | 0 | 0 | 0 | 0 | 0.07 | 0 | 0 | 0.496 | 0.511 |
| Cyp2e1 | Tp53 | 0 | 0 | 0 | 0 | 0 | 0 | 0 | 0.524 | 0.524 |
| Cyp2e1 | Gpx1 | 0 | 0 | 0 | 0 | 0.073 | 0 | 0 | 0.587 | 0.6 |
| Cyp2e1 | Hmox1 | 0 | 0 | 0 | 0 | 0.139 | 0 | 0 | 0.637 | 0.674 |
| Cyp2e1 | Gstm1 | 0 | 0 | 0 | 0 | 0 | 0.044 | 0.65 | 0.6 | 0.854 |
| Cyp2e1 | Gstp1 | 0 | 0 | 0 | 0 | 0 | 0.044 | 0.65 | 0.605 | 0.856 |
| Cyp2e1 | Hsd3b6 | 0 | 0 | 0 | 0 | 0.059 | 0.131 | 0.91 | 0.18 | 0.931 |
| Cyp1a2 | Nfe2l2 | 0 | 0 | 0 | 0 | 0 | 0 | 0 | 0.426 | 0.426 |
| Cyp1a2 | Cyp2e1 | 0 | 0 |  | 0.767 | 0.171 | 0 | 0.9 | 0.9 | 0.938 |
| Cyp1a2 | Hmox1 | 0 | 0 | 0 | 0 | 0.139 | 0 | 0 | 0.435 | 0.493 |
| Cyp1a2 | Tp53 | 0 | 0 | 0 | 0 | 0 | 0 | 0 | 0.414 | 0.414 |
| Cyp1a2 | Hsd3b6 | 0 | 0 | 0 | 0 | 0.064 | 0.131 | 0.685 | 0.064 | 0.728 |
| Cyp1a2 | Gstm1 | 0 | 0 | 0 | 0 | 0.08 | 0.044 | 0.65 | 0.605 | 0.862 |
| Cyp1a2 | Gstp1 | 0 | 0 | 0 | 0 | 0 | 0.044 | 0.65 | 0.631 | 0.865 |
| Cyp1a2 | Cyp1b1 | 0 | 0 | 0 | 0.815 | 0.309 | 0 | 0.8 | 0.881 | 0.879 |
| Cyp1a1 | Nfe2l2 | 0 | 0 | 0 | 0 | 0 | 0 | 0 | 0.527 | 0.527 |
| Cyp1a1 | Ptgs2 | 0 | 0 | 0 | 0 | 0.056 | 0.042 | 0.093 | 0.507 | 0.541 |
| Cyp1a1 | Cyp2e1 | 0 | 0 |  | 0.793 | 0.092 | 0 | 0.9 | 0.897 | 0.929 |
| Cyp1a1 | Hmox1 | 0 | 0 | 0 | 0 | 0.139 | 0 | 0 | 0.58 | 0.623 |
| Cyp1a1 | Cyp1a2 | 0 | 0 |  | 0.972 | 0.062 | 0 | 0.8 | 0.895 | 0.811 |
| Cyp1a1 | Gstp1 | 0 | 0 | 0 | 0 | 0 | 0.044 | 0.65 | 0.702 | 0.891 |
| Cyp1a1 | Hsd3b6 | 0 | 0 | 0 | 0 | 0.058 | 0.131 | 0.685 | 0.257 | 0.783 |
| Cyp1a1 | Gpx1 | 0 | 0 | 0 | 0 | 0 | 0 | 0 | 0.441 | 0.441 |
| Cyp1a1 | Tp53 | 0 | 0 | 0 | 0 | 0 | 0 | 0 | 0.584 | 0.584 |
| Cyp1a1 | Gstm1 | 0 | 0 | 0 | 0 | 0 | 0.044 | 0.65 | 0.674 | 0.881 |
| Cyp1a1 | Cyp1b1 | 0 | 0 | 0 | 0.842 | 0.309 | 0 | 0.9 | 0.932 | 0.938 |
| Cyp1a1 | Hsd17b1 | 0 | 0 | 0 | 0 | 0 | 0.057 | 0.913 | 0.404 | 0.947 |
| Gstm1 | Nfe2l2 | 0 | 0 | 0 | 0 | 0.062 | 0 | 0.323 | 0.522 | 0.669 |
| Gstm1 | Sod1 | 0 | 0 | 0 | 0 | 0.326 | 0 | 0 | 0.304 | 0.511 |
| Gstm1 | Jun | 0 | 0 | 0 | 0 | 0 | 0 | 0.319 | 0.206 | 0.436 |
| Gstm1 | Hmox1 | 0 | 0 | 0 | 0 | 0 | 0 | 0 | 0.572 | 0.572 |
| Gstm1 | Gstp1 | 0 | 0 | 0 | 0.789 | 0.057 | 0 | 0.65 | 0.878 | 0.719 |
| Gstp1 | Nfe2l2 | 0 | 0 | 0 | 0 | 0 | 0 | 0.323 | 0.614 | 0.728 |
| Gstp1 | Sod1 | 0 | 0 | 0 | 0 | 0.064 | 0.054 | 0 | 0.454 | 0.474 |
| Gstp1 | Ptgs2 | 0 | 0 | 0 | 0 | 0 | 0 | 0.136 | 0.394 | 0.454 |
| Gstp1 | Jun | 0 | 0 | 0 | 0 | 0 | 0.045 | 0.319 | 0.726 | 0.806 |
| Gstp1 | Hmox1 | 0 | 0 | 0 | 0 | 0 | 0 | 0 | 0.59 | 0.59 |
| Gstp1 | Sod2 | 0 | 0 | 0 | 0 | 0.064 | 0 | 0 | 0.424 | 0.438 |
| Gstp1 | Tp53 | 0 | 0 | 0 | 0 | 0 | 0 | 0 | 0.619 | 0.619 |
| Gstp1 | Mapk8 | 0 | 0 | 0 | 0 | 0 | 0.42 | 0 | 0.456 | 0.671 |
| Cdkn1c | Jun | 0 | 0 | 0 | 0 | 0.051 | 0.06 | 0.366 | 0.286 | 0.542 |
| Cdkn1c | Nr4a2 | 0 | 0 | 0 | 0 | 0 | 0.199 | 0 | 0.482 | 0.567 |
| Cdkn1c | Tp53 | 0 | 0 | 0 | 0 | 0 | 0.474 | 0.442 | 0.561 | 0.86 |
| Cdkn1c | Mapk8 | 0 | 0 | 0 | 0 | 0 | 0.122 | 0.366 | 0.096 | 0.453 |
| Cebpb | Mapk1 | 0 | 0 | 0 | 0 | 0 | 0.307 | 0.961 | 0.271 | 0.978 |
| Cebpb | Ptgs2 | 0 | 0 | 0 | 0 | 0.12 | 0.114 | 0 | 0.519 | 0.592 |
| Cebpb | Fos | 0 | 0 | 0 | 0 | 0.295 | 0.163 | 0.514 | 0.635 | 0.881 |
| Cebpb | Jun | 0 | 0 | 0 | 0 | 0.176 | 0.16 | 0.629 | 0.713 | 0.916 |
| Cebpb | Creb1 | 0 | 0 | 0 | 0 | 0 | 0.175 | 0 | 0.731 | 0.768 |
| Cebpb | Egr1 | 0 | 0 | 0 | 0 | 0.064 | 0.218 | 0.14 | 0.781 | 0.843 |
| Cebpb | Mapk3 | 0 | 0 | 0 | 0 | 0 | 0.193 | 0.959 | 0.481 | 0.981 |
| Cebpb | Nfkb1 | 0 | 0 | 0 | 0 | 0.06 | 0.114 | 0 | 0.601 | 0.638 |
| Cebpb | Rela | 0 | 0 | 0 | 0 | 0.064 | 0.55 | 0 | 0.571 | 0.803 |
| Cebpb | Tp53 | 0 | 0 | 0 | 0 | 0 | 0.092 | 0 | 0.487 | 0.514 |
| Gh1 | Mapk1 | 0 | 0 | 0 | 0 | 0 | 0.042 | 0.9 | 0.081 | 0.904 |
| Gh1 | Mapk3 | 0 | 0 | 0 | 0 | 0 | 0.043 | 0.9 | 0.105 | 0.906 |
| Gh1 | Prl | 0 | 0 | 0 | 0.668 | 0.424 | 0.124 | 0.8 | 0.764 | 0.917 |
| Lhb | Slc18a2 | 0 | 0 | 0 | 0 | 0 | 0 | 0 | 0.448 | 0.448 |
| Lhb | Prl | 0 | 0 | 0 | 0 | 0.119 | 0 | 0 | 0.462 | 0.506 |
| Lhb | Th | 0 | 0 | 0 | 0 | 0 | 0 | 0 | 0.608 | 0.608 |
| Hsd3b6 | Lhb | 0 | 0 | 0 | 0 | 0 | 0 | 0 | 0.473 | 0.473 |
| Cyp1b1 | Nfe2l2 | 0 | 0 | 0 | 0 | 0 | 0 | 0 | 0.425 | 0.424 |
| Cyp1b1 | Ptgs2 | 0 | 0 | 0 | 0 | 0.063 | 0.042 | 0.093 | 0.458 | 0.499 |
| Cyp1b1 | Hmox1 | 0 | 0 | 0 | 0 | 0.139 | 0 | 0 | 0.41 | 0.47 |
| Cyp1b1 | Gstp1 | 0 | 0 | 0 | 0 | 0 | 0.044 | 0.65 | 0.608 | 0.857 |
| Cyp1b1 | Hsd3b6 | 0 | 0 | 0 | 0 | 0 | 0.131 | 0.138 | 0.307 | 0.435 |
| Cyp1b1 | Hsd17b1 | 0 | 0 | 0 | 0 | 0 | 0.057 | 0.913 | 0.523 | 0.957 |
| Cyp1b1 | Gstm1 | 0 | 0 | 0 | 0 | 0 | 0.044 | 0.65 | 0.586 | 0.849 |
| Cyp1b1 | Tp53 | 0 | 0 | 0 | 0 | 0 | 0 | 0 | 0.508 | 0.508 |
| Hsd17b1 | Hsd3b6 | 0.043 | 0 | 0 | 0 | 0.064 | 0.12 | 0.145 | 0.759 | 0.808 |

**Table S3.** GO enrichment analysis

| **GO term** | **category** | **Count** | **%** | **Log10(P)** | **Log10(q)** |
| --- | --- | --- | --- | --- | --- |
| GO:0010038 | Biological Processes | 40 | 62.5 | -45.1 | -40.91 |
| GO:0009410 | Biological Processes | 39 | 60.94 | -38.6 | -34.7 |
| GO:0006979 | Biological Processes | 33 | 51.56 | -34.47 | -30.76 |
| GO:0001666 | Biological Processes | 26 | 40.62 | -24.67 | -21.52 |
| GO:1901652 | Biological Processes | 29 | 45.31 | -24.31 | -21.23 |
| GO:0048545 | Biological Processes | 24 | 37.5 | -21.64 | -18.72 |
| GO:1902074 | Biological Processes | 25 | 39.06 | -21.53 | -18.63 |
| GO:0035094 | Biological Processes | 14 | 21.88 | -20.34 | -17.51 |
| GO:1901214 | Biological Processes | 22 | 34.38 | -20.14 | -17.32 |
| GO:0046686 | Biological Processes | 14 | 21.88 | -19.53 | -16.73 |
| GO:0044306 | Cellular Components | 10 | 15.62 | -7.75 | -4.63 |
| GO:0044853 | Cellular Components | 8 | 12.5 | -7.61 | -4.63 |
| GO:0019867 | Cellular Components | 8 | 12.5 | -6.33 | -3.96 |
| GO:0032839 | Cellular Components | 4 | 6.25 | -5.01 | -2.96 |
| GO:0030061 | Cellular Components | 3 | 4.69 | -4.67 | -2.73 |
| GO:0000785 | Cellular Components | 11 | 17.19 | -4.51 | -2.61 |
| GO:0098978 | Cellular Components | 10 | 15.62 | -4.1 | -2.24 |
| GO:0048471 | Cellular Components | 9 | 14.06 | -2.85 | -1.15 |
| GO:0016234 | Cellular Components | 3 | 4.69 | -2.54 | -0.91 |
| GO:0036126 | Cellular Components | 4 | 6.25 | -2.45 | -0.84 |
| GO:0016491 | Molecular Functions | 19 | 29.69 | -11.07 | -7.38 |
| GO:0019901 | Molecular Functions | 17 | 26.56 | -9.33 | -5.94 |
| GO:0031072 | Molecular Functions | 9 | 14.06 | -8.54 | -5.33 |
| GO:0035240 | Molecular Functions | 4 | 6.25 | -8.34 | -5.25 |
| GO:0016209 | Molecular Functions | 7 | 10.94 | -7.91 | -5.02 |
| GO:0019902 | Molecular Functions | 9 | 14.06 | -7 | -4.38 |
| GO:0042803 | Molecular Functions | 14 | 21.88 | -6.82 | -4.24 |
| GO:0044389 | Molecular Functions | 10 | 15.62 | -6.67 | -4.12 |
| GO:0051434 | Molecular Functions | 3 | 4.69 | -6.1 | -3.71 |
| GO:0004712 | Molecular Functions | 4 | 6.25 | -5.2 | -2.98 |

**Table S4.** KEGG enrichment analysis

| **KEGG Pathway** | **category** | **Count** | **%** | **Log10(P)** | **Log10(q)** |
| --- | --- | --- | --- | --- | --- |
| rno05030 | KEGG Pathway | 13 | 20.31 | -21.44 | -18.9 |
| rno05022 | KEGG Pathway | 23 | 35.94 | -20.01 | -17.77 |
| rno05200 | KEGG Pathway | 23 | 35.94 | -18.84 | -16.78 |
| rno04024 | KEGG Pathway | 17 | 26.56 | -18.02 | -16.22 |
| rno05418 | KEGG Pathway | 12 | 18.75 | -12.88 | -11.65 |
| rno04913 | KEGG Pathway | 9 | 14.06 | -12.43 | -11.25 |
| rno05204 | KEGG Pathway | 7 | 10.94 | -8.11 | -7.33 |
| rno04918 | KEGG Pathway | 6 | 9.38 | -6.71 | -6.03 |
| rno04068 | KEGG Pathway | 7 | 10.94 | -6.4 | -5.75 |
| rno00140 | KEGG Pathway | 6 | 9.38 | -6.35 | -5.72 |
| rno04310 | KEGG Pathway | 6 | 9.38 | -4.6 | -4.1 |
| rno04080 | KEGG Pathway | 8 | 12.5 | -4.24 | -3.74 |
| rno04630 | KEGG Pathway | 5 | 7.81 | -3.62 | -3.17 |
| rno04141 | KEGG Pathway | 5 | 7.81 | -3.54 | -3.09 |
| rno00350 | KEGG Pathway | 3 | 4.69 | -3.51 | -3.08 |
| rno00330 | KEGG Pathway | 3 | 4.69 | -3.13 | -2.72 |
| rno04020 | KEGG Pathway | 4 | 6.25 | -2.03 | -1.69 |

**Table S5.** Top 10 targets calculated by MNC methods

| **Rank** | **Name** | **Score** |
| --- | --- | --- |
| 1 | Tp53 | 39 |
| 2 | Creb1 | 36 |
| 3 | Mapk3 | 36 |
| 4 | Casp3 | 35 |
| 5 | Fos | 34 |
| 6 | Cat | 34 |
| 7 | Hmox1 | 34 |
| 8 | Ptgs2 | 32 |
| 9 | Bdnf | 31 |
| 10 | Jun | 30 |

**Table S6.** Top 10 targets calculated by Closeness method

| **Rank** | **Name** | **Score** |
| --- | --- | --- |
| 1 | Tp53 | 50.5 |
| 2 | Creb1 | 48.66666667 |
| 3 | Mapk3 | 48.66666667 |
| 4 | Casp3 | 48.16666667 |
| 5 | Fos | 47.83333333 |
| 6 | Cat | 47.66666667 |
| 7 | Hmox1 | 47.66666667 |
| 8 | Ptgs2 | 46.66666667 |
| 9 | Bdnf | 46.16666667 |
| 10 | Jun | 45.66666667 |

**Table S7.** Top 10 targets calculated by MCC method

| **Rank** | **Name** | **Score** |
| --- | --- | --- |
| 1 | Tp53 | 7904075514 |
| 2 | Casp3 | 7901243880 |
| 3 | Mapk3 | 7899291442 |
| 3 | Ptgs2 | 7848280824 |
| 5 | Hmox1 | 7841906664 |
| 5 | Casp9 | 7802823360 |
| 7 | Cat | 7801540644 |
| 7 | Bcl2l1 | 7368270408 |
| 7 | Sirt1 | 7333251864 |
| 7 | Nfe2l2 | 7318208880 |

**Table S8.** Top 10 targets calculated by EPC method

| **Rank** | **Name** | **Score** |
| --- | --- | --- |
| 1 | Tp53 | 21.899 |
| 2 | Casp3 | 21.337 |
| 3 | Creb1 | 21.309 |
| 4 | Mapk3 | 21.063 |
| 5 | Cat | 21.03 |
| 6 | Hmox1 | 20.59 |
| 7 | Fos | 20.442 |
| 8 | Ptgs2 | 20.156 |
| 9 | Bdnf | 20.142 |
| 10 | Jun | 19.826 |

**Table S9.** Top 10 targets calculated by Betweenness method

| **Rank** | **Name** | **Score** |
| --- | --- | --- |
| 1 | Tp53 | 307.8711039 |
| 2 | Mapk3 | 250.5518816 |
| 3 | Fos | 239.8562821 |
| 4 | Creb1 | 233.3878383 |
| 5 | Cat | 181.7453954 |
| 6 | Hmox1 | 163.1191482 |
| 7 | Bdnf | 151.2600261 |
| 8 | Ptgs2 | 134.4622724 |
| 9 | Th | 116.0362209 |
| 10 | Jun | 111.8457795 |

**Table S10.** Top 10 targets calculated by Radiality method

| **Rank** | **Name** | **Score** |
| --- | --- | --- |
| 1 | Tp53 | 3.709677419 |
| 2 | Creb1 | 3.629032258 |
| 3 | Mapk3 | 3.629032258 |
| 3 | Fos | 3.612903226 |
| 5 | Casp3 | 3.612903226 |
| 5 | Cat | 3.596774194 |
| 7 | Hmox1 | 3.596774194 |
| 7 | Ptgs2 | 3.564516129 |
| 7 | Bdnf | 3.548387097 |
| 10 | Jun | 3.532258065 |

**Table S11.** Molecular docking

| **Herbicide** | **Target** | **Docking score (kcal/mol)** |
| --- | --- | --- |
| ATR | Tp53 | -4.6 |
| ATR | Ptgs2 | -6.5 |
| ATR | Mapk3 | -6.2 |
| ATR | Cat | -6.5 |
| ATR | Hmox1 | -5.6 |
